# Regulation of *Drosophila* brain development and organ growth by the Minibrain/Rala signaling network

**DOI:** 10.1093/g3journal/jkae219

**Published:** 2024-09-13

**Authors:** Melissa Brown, Erika Sciascia, Ken Ning, Wesam Adam, Alexey Veraksa

**Affiliations:** Department of Biology, University of Massachusetts Boston, Boston, MA 02125, USA; Department of Biology, University of Massachusetts Boston, Boston, MA 02125, USA; Department of Biology, University of Massachusetts Boston, Boston, MA 02125, USA; Department of Biology, University of Massachusetts Boston, Boston, MA 02125, USA; Department of Biology, University of Massachusetts Boston, Boston, MA 02125, USA

**Keywords:** minibrain, Drosophila, Rala, DYRK1A, brain development, organ growth

## Abstract

The human dual specificity tyrosine phosphorylation regulated kinase 1A (DYRK1A) is implicated in the pathology of Down syndrome, microcephaly, and cancer; however the exact mechanism through which it functions is unknown. Here, we have studied the role of the *Drosophila* ortholog of DYRK1A, Minibrain (Mnb), in brain development and organ growth. The neuroblasts (neural stem cells) that eventually give rise to differentiated neurons in the adult brain are formed from a specialized tissue in the larval optic lobe called the neuroepithelium, in a tightly regulated process. Molecular marker analysis of *mnb* mutants revealed alterations in the neuroepithelium and neuroblast regions of developing larval brains. Using affinity purification-mass spectrometry (AP-MS), we identified the novel Mnb binding partners Ral interacting protein (Rlip) and RALBP1 associated Eps domain containing (Reps). Rlip and Reps physically and genetically interact with Mnb, and the three proteins may form a ternary complex. Mnb phosphorylates Reps, and human DYRK1A binds to the Reps orthologs REPS1 and REPS2. Mnb also promotes re-localization of Rlip from the nucleus to the cytoplasm in cultured cells. Furthermore, Mnb engages the small GTPase Ras-like protein A (Rala) to regulate brain and wing development. This work uncovers a previously unrecognized role of Mnb in the neuroepithelium and defines the functions of the Mnb/Reps/Rlip/Rala signaling network in organ growth and neurodevelopment.

## Introduction

The kinase Minibrain (Mnb) and its ortholog dual specificity tyrosine phosphorylation regulated kinase 1A (DYRK1A) control the development of the brain and other tissues in *Drosophila* and mammals (Tejedor *et al.* 1995; Guimera *et al.* 1999; Degoutin *et al.* 2013; Yang *et al.* 2016). In *Drosophila,* both loss and gain of *mnb* function can result in abnormal development. Reduction of *mnb* function results in decreased brain and wing size, disrupted wing vein patterning, abnormal visual and food intake behavior, and defects in dendrite development (Tejedor *et al.* 1995; Hong *et al.* 2012; Degoutin *et al.* 2013; Ori-McKenney *et al.* 2016; Yang *et al.* 2016). Gain of Mnb is associated with morphological defects, a shortened lifespan, impaired neuronal function, and accelerated decline in motor performance (Kim *et al.* 2016; Lowe *et al.* 2019). Likewise, the homolog of Mnb, dual specificity tyrosine phosphorylation regulated kinase 1A (DYRK1A), is critical for mammalian development and exhibits both loss and gain of function phenotypes (Ahn *et al.* 2006; Dowjat *et al.* 2007; van Bon *et al.* 2011; Ji *et al.* 2015; Raveau *et al.* 2018; Arbones *et al.* 2019). In humans, the *DYRK1A* gene is located on chromosome 21, and through studies in mouse models, *DYRK1A* copy number has been shown to play a causative role in Down syndrome neurodevelopmental defects (Ahn *et al.* 2006; Garcia-Cerro *et al.* 2014). Loss of function mutations in *DYRK1A* that lead to decreased DYRK1A protein levels result in primary microcephaly and developmental delays, reminiscent of *mnb* mutant phenotypes (Ji *et al.* 2015; Luco *et al.* 2016; Raveau *et al.* 2018). Given its disease phenotypes, it is clear that DYRK1A plays a critical role in neuronal development and physiology. DYRK1A has also been implicated in the control of the cell cycle and cell proliferation. Depending on the cellular context, DYRK1A can either promote or inhibit cell proliferation, and has both oncogenic and tumor suppressor characteristics (Yabut *et al.* 2010; Litovchick *et al.* 2011; Fernandez-Martinez *et al.* 2015; Rammohan *et al.* 2022).

*Drosophila* is a useful model system to study brain development, given the availability of genetic tools and similarities in neurogenesis between flies and mammals. In both *Drosophila* and mammalian brain development, there are stem cell populations that divide symmetrically to amplify a progenitor population, which then transitions to asymmetric divisions (Matsuzaki and Shitamukai 2015; Holguera and Desplan 2018; Mishra *et al.* 2018). In the optic lobe of the fly larval brain, the symmetrically dividing population is the neuroepithelium (NE), which gives rise to asymmetrically dividing stem cells, known as the neuroblasts (NBs) (Egger *et al.* 2007; Li *et al.* 2013; Neriec and Desplan 2016; Hakes *et al.* 2018). The inner proliferation center (IPC) of the optic lobe generates the lobula and lobula plate neurons, and the outer proliferation center (OPC) gives rise to the lamina and medulla neurons (Neriec and Desplan 2016).

Mnb is required for the development of the optic lobes, as this region of the brain is the one most affected in *mnb* mutants (Tejedor *et al.* 1995). Previous work has suggested that Mnb promotes cell cycle exit and neuronal differentiation of asymmetrically dividing neuronal precursors by controlling the expression of a transcription factor network involving Asense (Ase), Prospero (Pros) and Deadpan (Dpn) (Shaikh *et al.* 2016; Shaikh and Tejedor 2018). These transcription factors converge to upregulate expression of the cyclin dependent kinase inhibitor 1B ortholog Dacapo (Dap). Reduction of *mnb* function disrupts NB divisions, induces cell cycle defects, and results in mis-specification of neuronal precursors, which ultimately leads to apoptosis and a decrease in the adult brain size (Shaikh *et al.* 2016; Shaikh and Tejedor 2018). Despite this evidence, the molecular mechanisms of how Mnb controls the formation and functions of symmetrically dividing neural stem cells are incompletely understood.

Here we uncovered a previously unknown early developmental role of Mnb in controlling the transition between the neuroepithelium and neuroblasts. Using affinity purification-mass spectrometry (AP-MS), we identified the protein interaction network of Mnb and characterized RALBP1 Eps domain containing (Reps) and Ral interacting protein (Rlip) as novel binding partners of Mnb. We show that Reps and Rlip function together with Mnb to regulate the growth and development of the brain and the wing. We also determined that Mnb phosphorylates Reps, and that the physical interaction between Mnb and Reps is conserved across their human orthologs, DYRK1A and REPS1/2. Lastly, we investigated a connection between Mnb and the small GTPase Ras-like protein A (Rala), whose function is closely related to that of Rlip, and found that Mnb and Rala jointly control brain and wing growth. Collectively, this work establishes a novel role of Mnb in the neuroepithelium and identifies the Mnb/Reps/Rlip/Rala signaling axis as a regulator of brain development and organ growth.

## Materials and methods

### Fly stocks

All *Drosophila* stocks were maintained on standard yeast-cornmeal-agar medium at 25°C or 18°C as indicated. Fly lines used in this study: *mnb^d419^* (Hong *et al.* 2012), *UAS-mnbRNAi* (Kyoto #7826R-3), *da-GAL4* (Wodarz *et al.* 1995), *MS1096-GAL4* (Capdevila and Guerrero 1994), *UAS-RepsRNAi1* (VDRC #24719), *UAS-RepsRNAi2* (VDRC #110704), *UAS-RlipRNAi* (VDRC #105976). The following lines were obtained from the Bloomington *Drosophila* stock center: *c855a-GAL4* (#6990) (Egger *et al.* 2007), *UAS-wRNAi* (#33762), *UAS-Rala*^S25N^ (#32094), *Dp(1;3)DC337* (#30441), *Dp(1;3)RC033* (#38493).

### Immunostaining and fluorescence microscopy

3rd instar larval brains were dissected in 1× PBS with 0.3% Triton X-100 (Sigma Aldrich) and fixed in 4% paraformaldehyde (Electron Microscopy Sciences) in 1× PBS for 20 minutes at room temperature (RT) in darkness. Brains were washed three times for 5 minutes at RT with 1× PBS before blocking for 30 minutes with 5% normal goat serum (Fisher) in 1× PBS with Triton X-100. Brains were incubated in primary antibody (rat anti D-Cad2, Developmental Studies Hybridoma Bank, 1:20, rabbit anti cleaved Dcp-1, Cell Signaling Technology, 1:100, or rat anti Miranda, Abcam, 1:500) diluted in 1× PBS 0.3% Triton X-100 overnight. To immunostain two genotypes in the same well, eye discs and the mouth hooks were removed from brains from one genotype and were kept attached in the other genotype. Following primary antibody incubation, brains were washed 3 times for 20 minutes with 1× PBS before incubation with secondary antibody (goat anti rat Alexa Fluor 555, Thermo, 1:500 or goat anti rabbit Alexa Flour 555, Thermo) diluted in 1× PBS 0.3% Triton X-100 for 3 hours. Brains were washed following secondary antibody 3 times for 20 minutes with 1× PBS before 10–15 brains per slide were mounted between two coverslips placed approximately 0.5 cm apart in 15 µL of ProLong Gold Antifade Mountant, With DAPI (Fisher).

Fluorescent images were acquired using a Zeiss LSM 880 confocal microscope at 40× objective with 1 Airy Unit pinhole. Z-stacks were acquired with optimal slice numbers and maximum-intensity projections were performed in Zen Blue software. Arrowheads and neuroepithelium-neuroblast boundary lines were added in Adobe Illustrator 2023. Corrected total fluorescence was calculated as: raw intensity of Dcp-1 in optic lobe—(average background intensity × optic lobe area) in Fiji (Schindelin *et al.* 2012).

### Affinity purification from embryos and mass spectrometry analysis

Flies from the Mnb-TagRFP-T line (Yang *et al.* 2016) and *yw* controls were set up in 5 L fly cages with apple juice plates. Flies were allowed to lay eggs for 15 hours at RT, then apple juice plates containing embryos were aged for 3 hours at 25°C. Approximately 1 g of embryos were dechorionated with 50% bleach for 1.5 minutes and washed with water. Embryos were then lysed on ice with 4 mL of ice-cold default lysis buffer (DLB) (50 mM Tris (pH 7.5), 5% glycerol, 0.2% IGEPAL, 1.5 mM MgCl_2_, 125 mM NaCl, 25 mM NaF, 1 mM Na_3_VO_4_, with 2× cOmplete Protease Inhibitor, MilliporeSigma, 1 tablet per 25 mL of lysis buffer) with additional IGEPAL added to a final concentration of 0.5%, in a glass homogenizer using 30 strokes with a tight pestle. The lysates were incubated on ice for 20 minutes and then centrifuged at 16,000 rcf for 20 minutes at 4°C. The supernatant was then filtered through a pre-chilled 0.45 µm filter (Fisher). The filtered supernatant was then incubated with 50 µL of packed Pierce Control Agarose beads (Thermo) at 4°C for 30 minutes with rotation. The lysate was then incubated with 20 µL of packed RFP-trap Agarose beads (Bulldog Bio) for 2 hours at 4°C with rotation. After binding, the beads were washed 5 times with 1 mL of DLB with 0.5% IGEPAL. Then 40 µL of 4× SDS sample buffer were added to beads and the samples were heated at 95°C for 6 minutes. Samples were analysed on a NuPAGE 4–12% Bis-Tris Protein Gel (Fisher) and SilverQuest Silver Staining Kit (Fisher) to confirm sample quality. Protein samples for mass spectrometry were prepared by running 1 cm into an 8% Tris-Glycine SDS-PAGE gel followed by Coomassie blue staining. Two gel pieces (>75 kDa and <75 kDa) were cut from the gel for each sample and sent for mass spectrometry (MS) analysis. The MS analysis was conducted on two biological replicates for experimental samples (Mnb-TagRFP-T) and three control samples (*yw*).

Samples were analysed by liquid chromatography/tandem mass spectrometry (nanoLC-MS/MS) using Thermo Scientific Orbitrap mass spectrometer by the Taplin Mass Spectrometry Facility at Harvard Medical School. Mass spectrometry data were analysed using Sequest and searches were run using a database of annotated *Drosophila* proteins, with the following search parameters: mass tolerance = 2, mass units = amu, fragment ion tolerance = 1, ion series = nB;nY;b;y, Mods = 15.9949146221 M 14.0157 C, enzyme = trypsin. MS results from two gel pieces for each sample were combined by summing unique peptide counts for each protein. The results for experimental and control samples were compared using the Significance Analysis of INTeractome (SAINT) (v2.5.0) (Choi *et al.* 2011) using peptide counts identified for each protein. Any protein with the SAINT score (AvgP) above 0.8 was considered a high-confidence interactor and was included in the interactome (Supplementary File 1).

The Mnb protein interactome was generated using the STRING database app in Cytoscape (Shannon *et al.* 2003) using the following standard options: Network type: Full STRING Network, Confidence Score (Cutoff): 0.40, Maximum additional interactors: 0, Use smart delimiters: checked. The resulting STRING network was clustered using the clusterMaker MCL Cluster app in Cytoscape. The following MCL cluster settings were used: Granularity parameter: 2.5, Array Source: None, Edge cut off: 0, Assume edges are undirected: check, Adjust loops before clustering: check, Create new clustered network: check. To analyze Gene Ontology of the resulting clustered network, highly interconnected clusters were analysed using STRING enrichment.

### Plasmid construction

pMT-Reps-Flag and pMT-Reps-HA were generated by cloning Reps-RA isoform from FMO04419 (DGRC) into pMT-V5-His vector (Invitrogen). pMT-Mnb-V5 was generated by cloning Mnb-RH isoform from FMO12028 (DGRC) into pMT-V5-His vector. pMT-Rlip-HA was generated by cloning the Rlip-RA isoform from GH01995 (DGRC) into pMT-V5-His vector. pMT-mCherry-Rlip and pMT-Rlip-mCherry were cloned from pMT-Rlip-HA using NEB HiFi Assembly kit (NEB) to exclude the retained intron and incorporate the mCherry open reading frame into pMT-V5-His vector. pcDNA3.1-DYRK1A-V5 was generated by cloning DYRK1A from pMH-SFB-DYRK1A (Addgene) into pcDNA3.1(−) (ThermoFisher). pcDNA3.1-REPS2-Flag was obtained from Sinobiological. pcDNA3.1-REPS1-Flag was generated by cloning REPS1 from pcMV-REPS1 (Sinobiological) into pcDNA3.1(−).

### Co-immunoprecipitation (co-IP)

*Drosophila* S2 cells were transfected with the indicated plasmids or blank pMT-V5-His vector (Invitrogen) using Effectene transfection reagent (Qiagen). 24 hours after transfection, cells were induced with 0.35 mM CuSO_4_ overnight at 25°C. Cells were then collected, washed with 10 mL of 1× PBS (Fisher), and lysed on ice for 20 minutes with 700 µL of ice-cold DLB. The lysate was centrifuged at 20,000 rcf for 20 minutes at 4°C. To generate lysate samples, 50 µL of lysate were added to 25 µL of 4× SDS sample buffer and heated for 5 minutes at 95°C. 450 µL of the lysate were incubated with 20 µL of packed anti-V5 beads (MilliporeSigma), anti-Flag beads (MilliporeSigma), anti-HA beads (MilliporeSigma), or anti-RFP beads (Bulldog Bio) for 2 hours at 4 °C. The beads were then washed 5 times with 1 mL of DLB. 40 µL of 4× SDS sample buffer was added and the beads were incubated at 95°C for 5 minutes to generate IP samples. The lysate and IP samples were run on SDS-PAGE followed by western blot.

### Phos-tag analysis and western blotting

Samples were prepared using EDTA-free DLB (composition as above, except with 2× cOmplete EDTA-free Protease Inhibitor, MilliporeSigma, 1 tablet per 25 mL of lysis buffer) and immunoprecipitated with anti-Flag beads. IP sample was analyzed by standard SDS-PAGE and Phos-tag analysis. A 6% Phos-tag gel with 50 µM Phos-tag (FujiFilm Wako Chemicals) and 100 µM MnCl_2_ was run at 80 V for 4 hours at 4°C. Following electrophoresis, the Phos-tag gel was washed with 10 mM EDTA in 1× Transfer Buffer 2 times for 10 minutes followed by one wash with 1× Transfer Buffer for 10 minutes. The gel was then wet transferred at 90 V for 2 hours at 4°C. The blots were then blocked in blocking buffer (LI-COR) for 30 minutes before incubation with primary antibody (rabbit anti-FLAG, Sigma-Aldrich, 1:1000) and secondary antibody donkey anti-rabbit IgG (1:20,000, LI-COR). Blots were scanned on LI-COR Odyssey CLx Imaging System. Other antibodies used for western blotting were mouse anti-V5 (MilliporeSigma, 1:1000), rabbit anti-HA (MilliporeSigma, 1:1000), rabbit anti-dsRed (Takara, 1:1000), and Goat anti-Mouse IgG (LI-COR, 1:20,000).

### Wing and brain analysis

Adult female wings were removed and mounted in 3:1 CMCP-10 Mounting Medium (Fisher)/lactic acid. Wing images were taken with a Zeiss Axiocam 712 camera on an Olympus BX60 Microscope. The wing size was calculated as the wing area and analyzed using Fiji. Adult female heads were removed and fixed in 4% paraformaldehyde in 1× PBS for 20 minutes at RT in darkness. Following fixation, heads were washed 3 times in 1× PBS for 5 minutes at RT. Brains were dissected from fixed heads before mounting. 10–15 brains per slide were mounted between two coverslips placed approximately 0.5 cm apart in 15 µL of ProLong Gold Antifade Mountant, With DAPI (Fisher). Slides were stored overnight in darkness at RT before sealing the edges with clear nail polish. Fluorescent images of brains were taken with a Zeiss Axiocam 712 camera on an Olympus BX60 Microscope with a 10× objective. Brain size was measured in Fiji as the area of the DAPI positive brain tissue.

## Results

### Mnb regulates neuroepithelium development

**Fig. 1.**
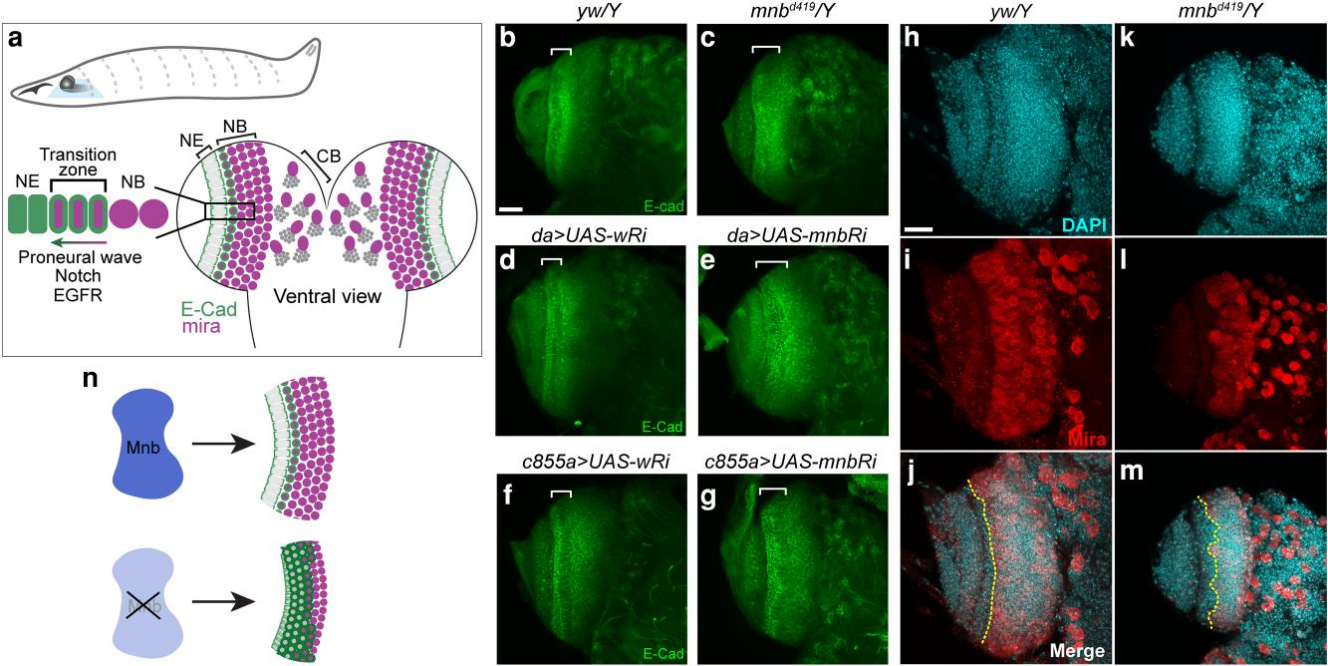
Mnb is required for neuroepithelium and neuroblast organization. a) Top: overall view of third instar larva with central nervous system shown. A horizontal square indicates imaging plane (ventral view). Bottom: Neuroepithelium (NE) to neuroblast (NB) transition is mediated by Notch and EGFR signaling. CB, central brain. Adapted from (Homem *et al.* 2015; Contreras *et al.* 2018). b-g) Confocal maximum intensity projections of larval brains of the indicated genotypes immunostained for E-cad. Brackets indicate the E-cad expression domain corresponding to the NE. RNAi (*Ri*) for the *white* gene (*wRi*) was used as a control in knockdown experiments. *da*, *da-GAL4* driver; *c855a*, *c855a-GAL4* driver. h-m) Confocal maximum intensity projections of larval brains of the indicated genotypes immunostained for Miranda (Mira), with the DAPI signal showing the DNA. Dotted line marks a boundary between the NE and the NB. Scale bars, 25 µm. n) Schematic representing NE/NB outcomes in wild type and *mnb* mutant conditions.

Previous work investigating the role of Mnb in larval brain development primarily focused on neuroblasts and their progeny in the central brain (CB) (Shaikh *et al.* 2016; Shaikh and Tejedor 2018). Given the significant size reduction of the adult optic lobes observed in *mnb* mutants (Tejedor *et al.* 1995; Yang *et al.* 2016), we investigated Mnb's role during the larval stages of optic lobe development. The optic lobes originate from NBs that are in turn produced from the neuroepithelial (NE) cells encompassing the inner and outer proliferation centers (Fig. 1a) (Hofbauer and Campos-Ortega 1990; Li *et al.* 2013). The NE cells must undergo a highly regulated transition from NE to NB, which is mediated by a proneural wave that is controlled by various signaling pathways including EGFR and Notch (Fig. 1a) (Yasugi *et al.* 2010; Jorg *et al.* 2019).

We used the mutant allele *mnb^d419^* (Hong *et al.* 2012) which deletes part of the kinase domain in the Mnb protein (Supplementary Fig. 1d) and results in smaller 3rd instar larval brains in hemizygous males (Yang *et al.* 2016). These hemizygous larvae formed pharate adults that died during eclosion, which is a more severe phenotype than the one observed for the kinase-inactive *mnb^1^* mutant allele that is homozygous viable (Tejedor *et al.* 1995; Ori-McKenney *et al.* 2016). Heterozygous female flies containing one copy of *mnb^d419^* and one copy of *mnb^1^* were viable (Supplementary Fig. 1). To ensure that *mnb^d419^* male lethality was not due to a mutation outside the *mnb* locus, we crossed *mnb^d419^* to two lines carrying duplications of the X chromosome containing the *mnb* gene and found that these lines rescued *mnb^d419^* lethality (Supplementary Fig. 2). These results demonstrate that the *mnb^d419^* allele is a strong loss of function mutation in *mnb*.

To visualize the NE, we stained for the epithelium marker E-cadherin (E-cad) (Simoes *et al.* 2017; Shard *et al.* 2020). *yw/Y* males were used as controls, and *mnb*^d419^*/Y* male larvae were used to analyze expression in the mutants. In control animals, E-cad staining revealed the typical NE organization with columnar epithelial cells expressing E-cad at their basal and apical sides (Fig. 1, b, d, and f, bracketed region). A few dividing epithelium cells can be identified as the rounded cells within the NE band and only a few cells within the NB region are E-cad positive. In contrast, the NE organization in *mnb^d419^* was disrupted, resulting in fewer columnar cells and expansion of E-cad staining into the NB region (Fig. 1c). Larval brains expressing *UAS-mnb* RNAi (*Ri*) under the control of the ubiquitous *da-GAL4* driver or the NE-specific driver *c855a-GAL4* showed a similar phenotype, compared to larval brains expressing *UAS-w* (*white*) RNAi as a control (Fig. 1d-g). These results suggest that Mnb is required for the proper organization and development of the NE in the optic lobe.

Given that NE cells give rise to NBs, we assessed the organization of NBs through staining for the NB marker Miranda (Mira) (Shen *et al.* 1997; Schuldt *et al.* 1998). Wild-type larval brains showed a distinct band of tightly packed Mira positive NBs (Fig. 1i). The boundary between Mira positive NBs and the Mira negative NE cells was straight and smooth in wild-type brains (Fig. 1h-j, dotted line). In contrast, the boundary between NE and NB in *mnb*^d419^*/Y* mutant brains was jagged and uneven (Fig. 1k-m), suggesting there may be Mira negative, E-cad positive cells persisting into the NB region. This disruption suggests a defect in NE to NB transition, a process that is tightly regulated by Notch and EGFR signaling (Contreras *et al.* 2018).

Increased apoptosis was observed in *mnb*^d419^*/Y* larval brains, based on cleaved Death caspase-1 (Dcp-1) staining (Supplementary Fig. 3), in agreement with previously reported results using other *mnb* mutants (Shaikh *et al.* 2016). Based on the significant increase in Dcp-1 intensity within the NB region and the mis-localization of the E-cad and Mira markers within this same region, we speculate that these cells are mis-specified and undergo programmed cell death, ultimately resulting in a smaller adult brain. Collectively, these data have uncovered a previously unknown early role of Mnb in regulating NE function (Fig. 1n).

### Reps and Rlip interact with Mnb

**Fig. 2.**
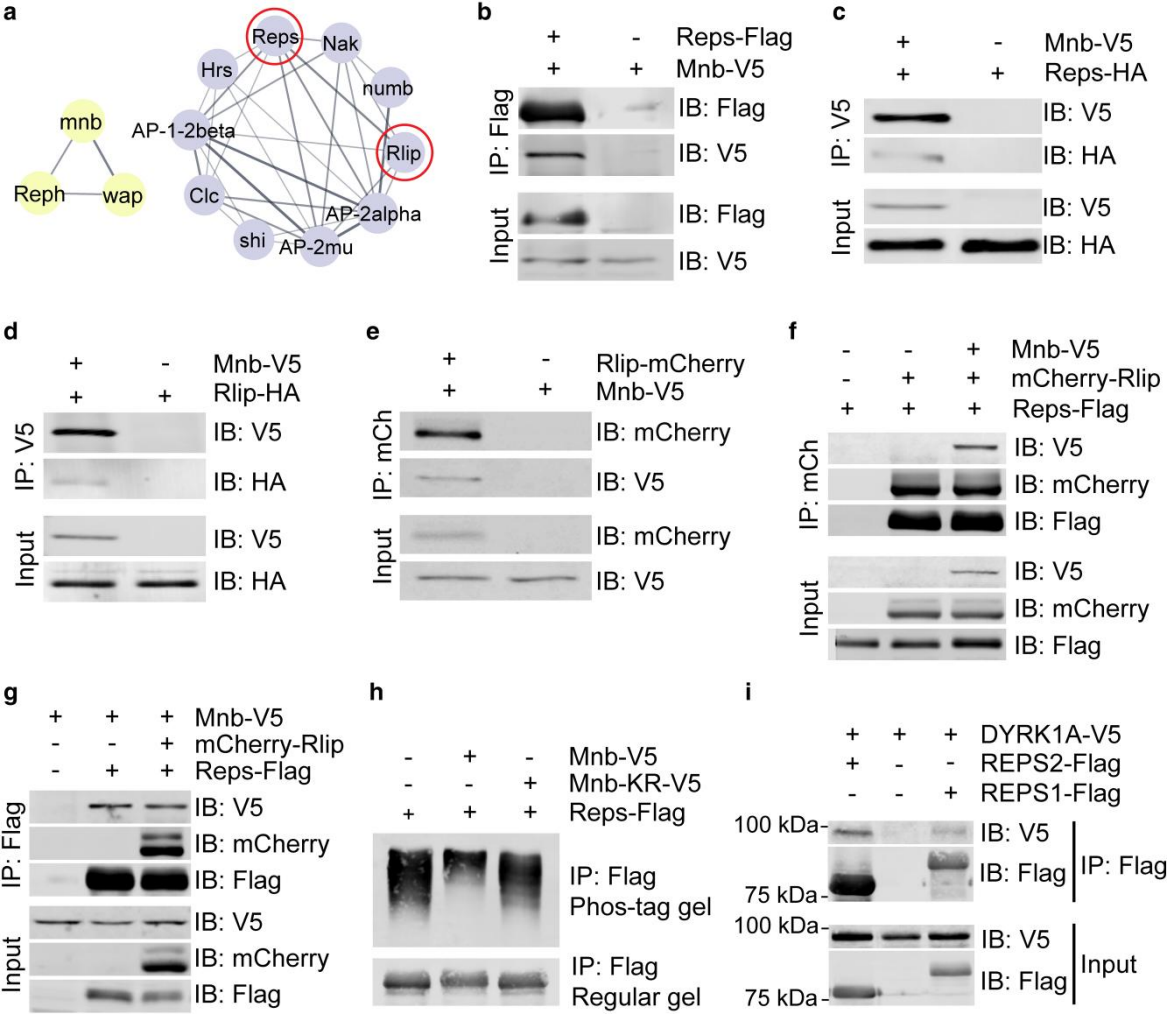
Mnb interacts with Reps and Rlip. a) Network clusters identified in Mnb AP-MS include known Mnb interactors Wap and Reph (left cluster) and a highly interconnected group of endocytosis related proteins (right cluster). b-g) Co-immunoprecipitation (co-IP) experiments in *Drosophila* S2 cells showing the binding interactions among Mnb, Reps, and Rlip using the indicated protein combinations and IP directions. Mnb showed pairwise interactions with Reps (b, c) and Rlip (d, e), and a ternary interaction with Reps and Rlip (f, g). h) Phos-tag analysis of Reps phosphorylation by Mnb. Bottom: regular SDS/PAGE gel. Mnb-KR, a kinase dead version of Mnb (Mnb^K193R^ mutant) (Degoutin *et al.* 2013). i) Co-IP of DYRK1A and REPS1/2 in HEK293T cells through IP of REPS1/2. IP: immunoprecipitation, IB: immunoblot.

To further characterize the mechanisms through which Mnb regulates brain development, we identified Mnb interacting proteins using AP-MS in embryos from endogenously tagged Mnb-TagRFP-T (Yang *et al.* 2016) and control flies. Peptide counts were analyzed by Significance Analysis of INTeractome (SAINT), and proteins with interaction probability score above 0.8 were considered significant (Supplementary File 1) (Choi *et al.* 2011). The resulting interaction network of significant hits was generated in Cytoscape using the STRING protein interaction database and clustered using the clusterMaker MCL Cluster app (Supplementary Fig. 4, see Materials and Methods) (Szklarczyk *et al.* 2015). Apart from Mnb itself, one of the top hits was the known Mnb interactor Wings apart (Wap) (Degoutin *et al.* 2013; Yang *et al.* 2016) (Fig. 2a). We also found Regulator of eph (Reph) which was previously identified as a Mnb interactor in the *Drosophila* Protein Interaction Map (Guruharsha *et al.* 2011) (Fig. 2a). Identification of these known interactors validates our AP-MS approach. Gene Ontology (GO) analysis of clusters showed several significantly enriched categories such as nuclear import (FDR = 4.9E−13), ribosome biogenesis (FDR = 8.01E−21), and endocytosis (FDR = 1.64E−8) (Supplementary Fig. 4).

The highly enriched complex of endocytic regulators (Fig. 2a) was interesting due to its potential impact on signaling, and we pursued characterization of two novel Mnb interactors from that cluster, RALBP1 associated Eps domain containing (Reps) and Ral interacting protein (Rlip). The mammalian orthologs of these proteins, REPS1/2 and RalA binding protein 1 (RALBP1), interact with activated small GTPase RalA and AP2 components to regulate receptor endocytosis (Ikeda *et al.* 1998; Nakashima *et al.* 1999; Jullien-Flores *et al.* 2000). RALBP1 also has mitosis-specific roles and serves as an essential adaptor between Cdk1 and Epsin, enabling Cdk1-mediated phosphorylation of Epsin which renders it endocytosis-incompetent and functions as a molecular off switch for endocytosis during mitosis (Rosse *et al.* 2003).

Co-immunoprecipitation (co-IP) of overexpressed tagged proteins confirmed the binding of Reps and Rlip to Mnb in *Drosophila* S2 cells (Fig. 2b-e). When expressed together, both Reps and Mnb can interact with Rlip, suggesting that these three proteins may form a ternary complex (Fig. 2f-g). Given the canonical role of Mnb as a kinase (Tejedor *et al.* 1995; Tejedor and Hammerle 2011; Yang *et al.* 2016), we investigated whether Mnb could phosphorylate Reps and Rlip. Phos-tag gel analysis of Reps revealed a mobility shift upon co-expression with wild type Mnb but not kinase dead Mnb (Fig. 2h), suggesting that Mnb is sufficient to phosphorylate Reps and Mnb kinase activity is required for this phosphorylation. These findings validate Reps and Rlip as bona fide Mnb protein interactors.

In mammals, REPS1/2 and RALBP1 also physically interact (Ikeda *et al.* 1998; Wang *et al.* 2023) but their interaction with the Mnb ortholog DYRK1A has not been reported. We found that REPS1 and REPS2 co-immunoprecipitated with DYRK1A in cultured HEK293T cells (Fig. 2i), whereas RALBP1 showed only a weak interaction with DYRK1A (Supplementary Fig. 5). These data show that the Mnb/Reps interaction is conserved, suggesting a possible involvement of DYRK1A in the regulation of REPS1/2 functions in human cells.

### Mnb promotes cytoplasmic Rlip localization

**Fig. 3.**
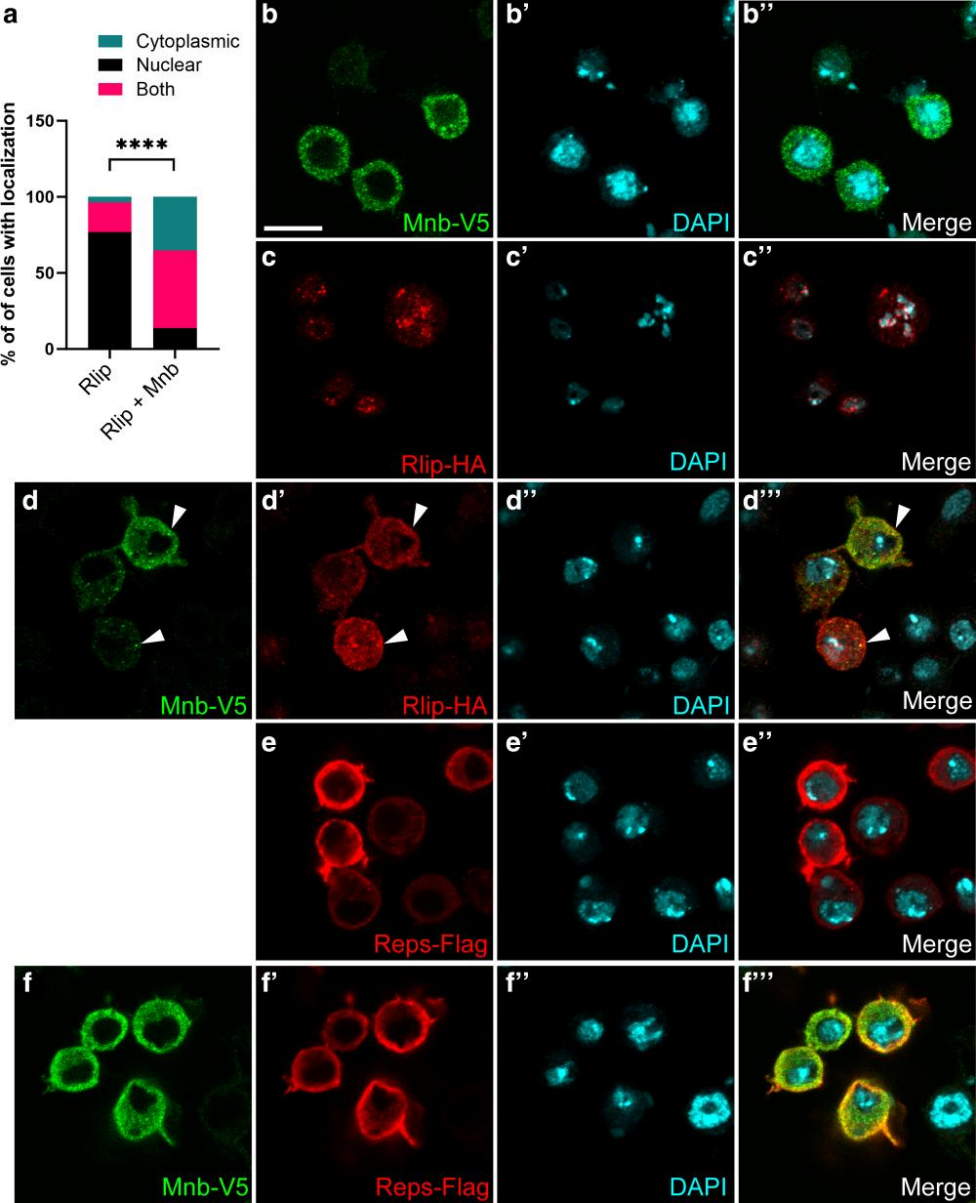
Mnb promotes cytoplasmic localization of Rlip. a) Quantification of data for (c-d‴). b-f‴) Confocal images of immunostained *Drosophila* S2 cells expressing Mnb-V5 (b-b″), Rlip-HA (c-c″), Mnb-V5 with Rlip-HA (d-d‴), Reps-Flag (e-e″), and Mnb-V5 with Reps-Flag (f-f‴). Arrowheads indicate Mnb-Rlip co-localization in cytoplasmic puncta (d-f‴). Scale bar in (b), 10 µm. Statistics calculated by chi-squared analysis, n > 100 for each condition, **** *P* ≤ 0.0001.

To probe the role of Mnb in the Mnb-Reps-Rlip complex, we investigated the localization patterns of these proteins in cultured *Drosophila* S2 cells. Mnb localized to the cytoplasm in a diffused pattern with a few distinct puncta in each cell (Fig. 3b-b″). Rlip was found mostly in the nucleus, where it formed puncta, but was also occasionally distributed throughout the cell (Fig. 3c-c″, quantified in Fig. 3a). Reps localized in a diffused cytoplasmic pattern (Fig. 3e-e″). Co-expression of Mnb and Rlip resulted in subcellular redistribution of Rlip from mostly nuclear to primarily cytoplasmic or uniform localization (Figs. 3d-d‴ and 3a). Mnb is therefore sufficient for retaining Rlip in the cytoplasm. This is significant because other Rlip interactors such as Rala are also localized in the cytoplasm, and Mnb may thus be promoting Rlip's interaction with those factors. Reps localization remained unchanged upon co-expression with Mnb (Fig. 3f-f‴).

### Reps and Rlip work together with Mnb to regulate wing growth

**Fig. 4.**
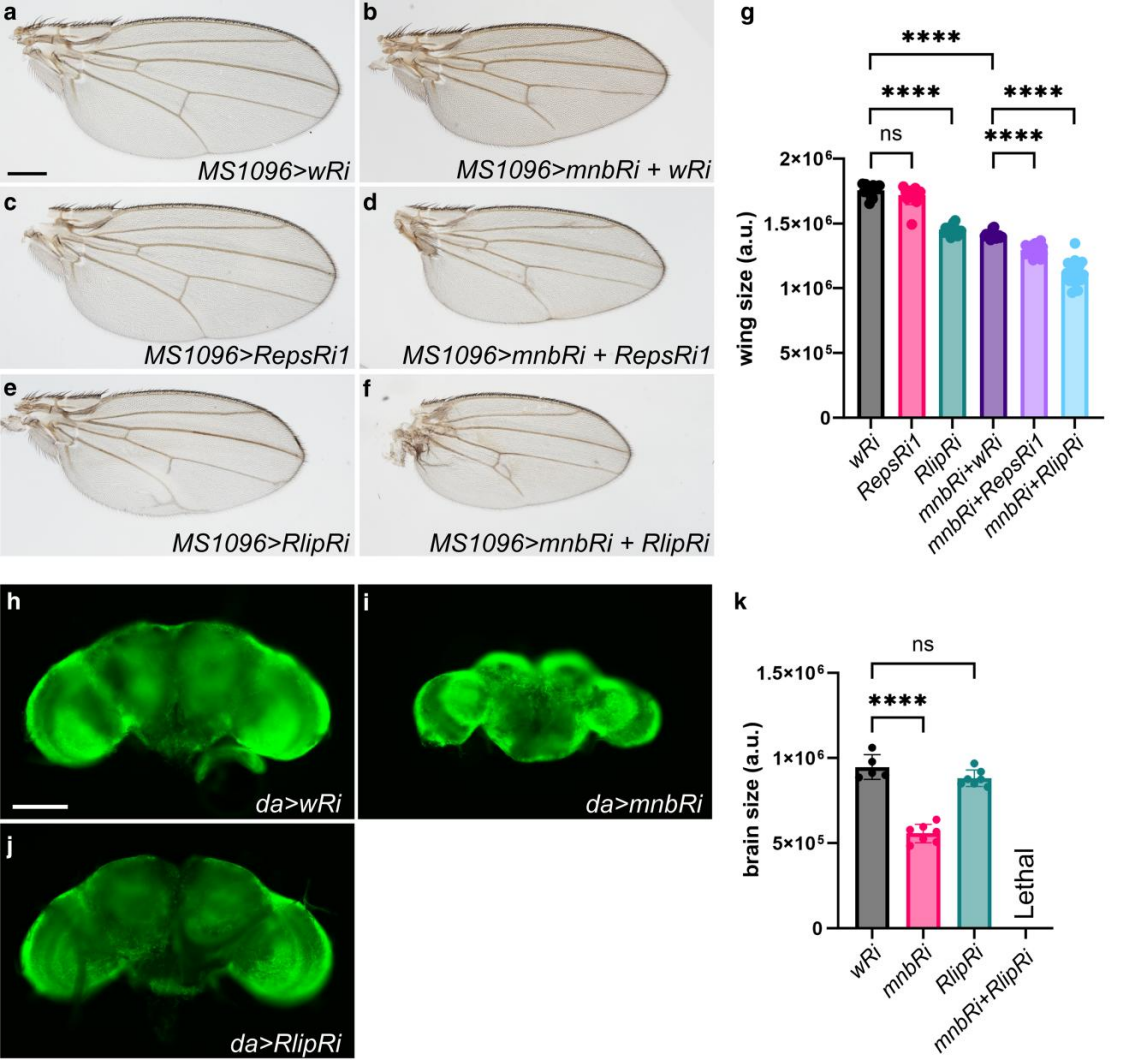
Mnb, Reps and Rlip work together to control wing growth and organism development. a-f) Wings from adult female flies expressing the indicated RNAi (*Ri*) transgenes using the wing-specific *MS1096-GAL4* driver. g) Quantification of the wing areas shown in (a-f) (n ≥ 14 for each genotype). ***P < 0.0001. *P* value calculated using ANOVA, error bars represent SD. Scale bar in (a), 300 µm. h-j) Adult brains from female flies grown at 18°C expressing the indicated RNAi transgenes using the ubiquitous *da-GAL4* driver. k) Quantification of brain size shown in (h-j) (*n* ≥ 5 for each genotype). Brain size was measured as total area. DAPI signal is shown in green. ****P < 0.0001, *P* value was calculated using ANOVA. Error bars represent SD. Scale bar in (H), 100 µm.

To determine whether Mnb, Reps, and Rlip are involved in common developmental processes, we tested the effects of knockdown of *Rlip* and *Reps* on the known *mnb* small wing phenotype. Knockdown of *mnb* with the wing-specific driver *MS1096-GAL4* resulted in a smaller wing than the control, consistent with previous observations (Degoutin *et al.* 2013; Yang *et al.* 2016) (Fig. 4, a, b and g). The knockdown of *Reps* did not alter wing growth but knockdown of *Rlip* resulted in a smaller wing than the control (Fig. 4, c, e and g). Double knockdown of either *Reps* or *Rlip* in conjunction with *mnb* led to a further reduction in wing size, compared to the knockdown of *mnb* alone (Fig. 4, d, f and g). These results suggest that Mnb, Reps, and Rlip work together to regulate wing growth.

### Rlip functions together with Mnb to regulate organism development

Given the critical role of Mnb in brain development (Tejedor and Hammerle 2011; Yang *et al.* 2016), we asked whether Reps and Rlip also regulate brain growth. Knockdown of *mnb* with the ubiquitously expressed driver *da-GAL4* led to a decrease in adult brain size, particularly in the optic lobes (Supplementary Fig. 6a, b). Knockdown of *Reps* with the same driver resulted in a larger brain than the control (Supplementary Fig. 6a, c), however double knockdown of *Reps* and *mnb* resulted in a brain that was similar in size to that of *mnb* knockdown alone (Supplementary Fig. 6a-e). Joint knockdown of *mnb* and *Reps* or *Rlip* with the NE-specific driver *c855a-GAL4* did not further decrease the size of the brain, compared to knockdown of *mnb* alone (Supplementary Fig. 7). These outcomes suggest that Mnb was epistatic to Reps and Rlip in these assays.

Using the *da-GAL4* driver, knockdown of either *Rlip* alone or *Rlip* in combination with *mnb* was lethal at 25°C. However, when shifted to 18°C, *RlipRi* flies were viable and did not show an altered brain size, compared to control flies also grown at 18°C (Fig. 4, j and k). *da-GAL4* driven knockdown of *mnb* at 18°C resulted in smaller brains than the control as expected (Fig. 4, h, i and k), however double knockdown of *mnb* and *Rlip* was lethal even at 18°C (Fig. 4k), suggesting a strong synergistic effect. The results of this experiment suggest that Mnb and Rlip function together to control *Drosophila* development, and the requirement for Rlip becomes apparent in the sensitized genetic background of reduced *mnb* function. Given the lethal phenotype of a joint knockdown of *mnb* and *Rlip* using a ubiquitously expressed driver, it is likely that their developmental functions are not limited to the brain.

Since reduction in *mnb* function results in the disruption of NE development (see Fig. 1), we asked whether a further reduction in *Reps* or *Rlip* would exacerbate this phenotype. A joint knockdown of *mnb* with *Reps* or *Rlip* using *da-GAL4* showed defects in E-cad staining that were similar to those observed for *mnb* alone (Supplementary Fig. 8). We note however that knockdown of *Rlip* alone also showed disorganization of the NE (Supplementary Fig. 8d), though this effect was not as strong as the phenotype obtained with knockdown of *mnb* (Supplementary Fig. 8b).

### Mnb engages Rala for growth control

**Fig. 5.**
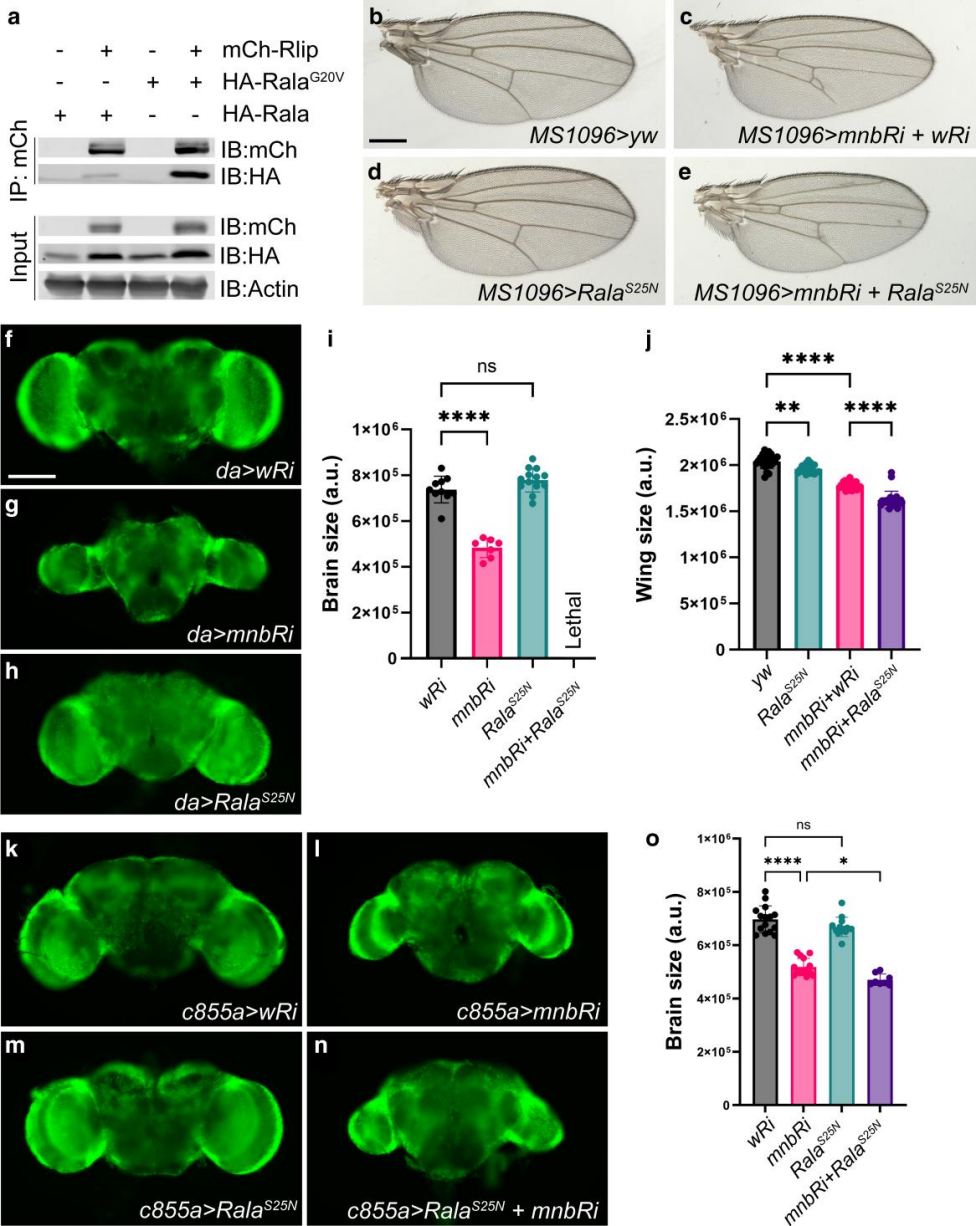
Mnb functions with Rala to regulate wing and brain development. a) Co-IP of Rlip, wild type Rala, and Rala^G20V^ in *Drosophila* S2 cells through IP of Rlip. Bb-e) Wings from adult female flies expressing the indicated transgenes using the *MS1096-GAL4* driver. A cross to *yw* (b) was used as a control. Scale bar in (b), 300 µm. f-h) Adult brains from female flies expressing the indicated RNAi (*Ri*) transgenes using the ubiquitous *da-GAL4* driver. Scale bar in (f), 100 µm. i) Quantification of the brain areas shown in (f-h) (*n*7 for each genotype). j) Quantification of the wing areas shown in (b-e) (*n*≥19 for each genotype). k-n) Adult brains from female flies expressing the indicated transgenes using the NE-specific *c855a-GAL4* driver. o) Quantification of brain size (area) shown in K-N (*n*≥8 for each genotype). ****P < 0.0001, **P < 0.001, *P < 0.01, *P* value calculated using ANOVA, error bars represent SD.

Rlip is a functional partner of the small GTPase Ras-like protein A (Rala) in the regulation of endocytosis and exocytosis of signaling receptors (Cantor *et al.* 1995; Nakashima *et al.* 1999; Wang *et al.* 2023). The Rlip ortholog preferentially interacts with activated GTP-bound Rala in *S. cerevisiae* (Cantor *et al.* 1995). We compared Rlip interactions with wild type Rala and a constitutively active variant Rala^G20V^ (Sawamoto *et al.* 1999). In *Drosophila* S2 cells, Rala^G20V^ bound to Rlip better than wild type Rala (Fig. 5a), confirming that *Drosophila* Rlip also preferentially interacts with activated Rala.

Given this connection between Rlip and Rala, we tested whether *Rala* and *mnb* interact genetically. We used a dominant-negative form of Rala (Rala^S25N^) that contains a serine to asparagine substitution at position 25 and as a result has a high affinity for GDP and cannot be activated (Sawamoto *et al.* 1999). In the wing, overexpression of Rala^S25N^ slightly reduced wing size (Fig. 5, b, d and j). Knockdown of *mnb* in conjunction with Rala^S25N^ overexpression reduced wing size more than either of these conditions alone (Fig. 5b-e, j). This suggests that Mnb and Rala cooperate to regulate wing growth. We also investigated the genetic interaction between Rala and Mnb in the adult brain. Interestingly, Rala^S25N^ overexpression phenocopied *Rlip-RNAi* in combination with *mnb-RNAi*: on its own, expression of Rala^S25N^ did not significantly alter adult brain size; however, when combined with *mnb* knockdown, this combination was lethal and adult brains could not be analyzed (Fig. 5f-i).

To determine whether Rala and Mnb function together in the NE, we used the NE-specific driver *c855a-GAL4* (Egger *et al.* 2007). Overexpression of Rala^S25N^ alone did not alter the brain size, while knockdown of *mnb* alone reduced the brain size (Fig. 5k-m). Importantly, combined Rala^S25N^ expression and *mnb* knockdown in the NE resulted in an even smaller brain than *mnb* knockdown alone (Fig. 5, n and o). Overall, these results suggest that Mnb and Rala jointly control the development of the wing and the brain, and in the brain their function is required specifically in the NE.

## Discussion

In this work, we have identified a previously unappreciated function of Mnb in the developing *Drosophila* brain. Mnb is required for proper organization of the NE, which harbors precursors of the neural stem cells (NBs), and for maintaining distinct cell fates in the NE and the NB regions. By using a strong loss of function allele, *mnb^d419^*, as well as NE-specific knockdowns, we show that loss of *mnb* disrupts NE integrity and compromises the boundary between the NE and the NBs, allowing aberrant expansion of E-cad positive cells into the NB region. The *mnb* small brain phenotype may therefore derive from increased apoptosis of mis-specified cells, resulting in fewer properly specified NBs and ultimately fewer neurons in the adult brain. Increased levels of apoptosis in *mnb* mutants have been reported previously (Shaikh *et al.* 2016), and we confirmed this observation for the *mnb^d419^* allele (Supplementary Fig. 3).

**Fig. 6.**
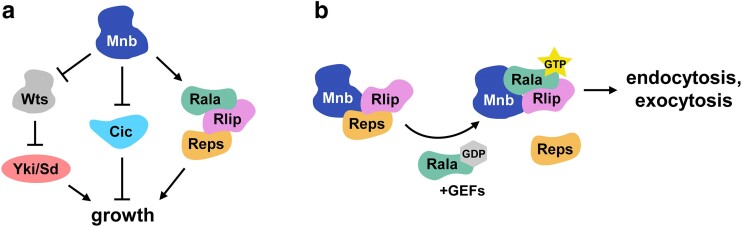
A model for the function of the Mnb/Reps/Rlip/Rala signaling network. a) Mnb participates in at least three regulatory pathways that control organ growth: Hippo/Warts (Wts)/Yorkie (Yki) (Degoutin *et al.* 2013), ERK/Cic (Yang *et al.* 2016), and the Reps/Rlip/Rala pathway described in this work. b) A possible molecular mechanism for Mnb-mediated control of brain development and organ growth. Mnb engages Reps and Rlip to activate Rala, which in turn controls receptor signaling via regulation of endo- and exocytosis. Reps is thought to dissociate from Rlip after Rala activation (Wang *et al.* 2023).

We identified a network of in vivo Mnb interacting proteins via AP-MS and uncovered several functional protein groups that may be targeted by or work together with Mnb. Among the proteins in a highly connected endocytosis related cluster, we identified Mnb interactors Reps and Rlip that may form a ternary complex with Mnb. Mnb/DYRK1A and Reps/REPS1/2 physically interact in both *Drosophila* and human cells, suggesting conservation of this interaction and a potential novel regulatory axis for DYRK1A-related diseases such as microcephaly. At the subcellular level, Mnb can recruit Rlip into the cytoplasmic puncta but did not affect Reps localization that was already cytoplasmic (Fig. 3). Beyond physical interactions, Mnb, Reps, and Rlip work together in controlling wing growth, and a ubiquitous joint knockdown of *mnb* and *Rlip* results in lethality, suggesting a more general common function in organism development. Given the functional connection between Rlip and Rala, we investigated a possible interaction between Mnb and Rala, and found that they similarly have a joint function in controlling wing and brain development. In the brain, the functions of Mnb and Rala are required specifically in the NE, suggesting that Rala contributes to Mnb activity in that tissue. Collectively, this evidence establishes a regulatory axis involving Mnb, Reps, Rlip, and Rala (Fig. 6a, b). These proteins may work together to regulate NE integrity and NB specification during brain development and are likely involved in the growth of other tissues.

We have previously established that Mnb contributes to the control of organ growth and tissue patterning in *Drosophila* via downregulation of the Hippo pathway kinase Warts (Degoutin *et al.* 2013) and via inactivation of the transcriptional repressor Capicua in parallel with ERK (Yang *et al.* 2016). Our current study delineates a third important signaling function of Mnb that involves trafficking regulators Reps, Rlip, and Rala (Fig. 6a). In the future, it would be of interest to determine how these pathways are coordinated to mediate proper development of the brain and other tissues.

It is possible that the Mnb/Reps/Rlip/Rala network controls signaling receptors at the level of intracellular trafficking. Rala is involved in the regulation of different signaling pathways such as c-Jun N-Terminal Kinase (JNK) (Sawamoto *et al.* 1999), EGFR/ERK (Douguet *et al.* 1988), and Notch (Cho and Fischer 2011), in various developmental contexts. The Rala-Rlip-Reps complex has been implicated in both endocytosis and exocytosis of signaling receptors (Nakashima *et al.* 1999; Wang *et al.* 2023). Given the central role for EGFR and Notch signaling in controlling NE development and orchestrating a proper propagation of the proneural wave in the transition to NBs (Egger *et al.* 2010; Yasugi *et al.* 2010; Contreras *et al.* 2018; Jorg *et al.* 2019), we speculate that the Mnb/Reps/Rlip/Rala signaling network contributes to the regulation of these pathways, possibly at the level of endo- or exocytosis (Fig. 6b). Another likely candidate for Mnb-mediated trafficking regulation is E-cad, whose subcellular localization is tightly controlled by both endo- and exocytosis (Bruser and Bogdan 2017). Consistent with this possibility, E-cad localization in the NE was disrupted in *mnb* mutants (Fig. 1 and Supplementary Fig. 8).

We have shown that Mnb can phosphorylate Reps and that this phosphorylation requires Mnb's kinase activity, but the significance of this effect is unclear. In S2 cells, the addition of Mnb does not reduce or enhance the Rlip-Reps interaction (Fig. 2). Therefore, we predict that Mnb phosphorylation of Reps has other mechanistic consequences outside of Mnb/Reps/Rlip interactions. It is possible that Mnb-mediated phosphorylation of Reps alters its interactions with Epsins which could affect recruitment of the Reps/Rlip complex to endocytic compartments (Morinaka *et al.* 1999; Kariya *et al.* 2000; Kim *et al.* 2021), thus regulating signaling. We note that Mnb is likely not the only kinase that phosphorylates Reps, because the Reps bands on a Phos-tag gel are not further slowed down with the addition of Mnb but rather the lower, hypo-phosphorylated forms of Reps gain additional phosphorylations (Fig. 2h). Given the role Reps plays as an adapter protein between Rlip/RALBP1 and Rala in mammalian cells (Wang *et al.* 2023), it is possible that it plays a similar role in *Drosophila*. A recent study found that mammalian REPS1 does not interact with RALA but instead helps stabilize RALBP1 until it interacts with RALA, and then Reps dissociates from the RALA-RALBP1 complex (Wang *et al.* 2023). The ability of Mnb to phosphorylate Reps and the formation of the Mnb/Reps/Rlip complex may contribute to a similar dynamic control of Reps/Rlip/Rala interactions in flies (Fig. 6b). Such dynamics may be critical for the proper regulation of signaling pathways responsible for the NE to NB transition, as well as for the development of other organ systems.

## Supplementary Material

jkae219_Supplementary_Data

## Data Availability

Strains and plasmids are available upon request. A complete SAINT output is provided in Supplementary File 1. The mass spectrometry proteomics data have been deposited to the ProteomeXchange Consortium via the PRIDE partner repository with the dataset identifier PXD052378. Supplemental material available at G3 online.

## References

[jkae219-B1] Ahn KJ , Jeong HK, Choi HS, Ryoo SR, Kim YJ, Goo JS, Choi SY, Han JS, Ha I, Song WJ. 2006. DYRK1A BAC transgenic mice show altered synaptic plasticity with learning and memory defects. Neurobiol Dis. 22(3):463–472. doi:10.1016/j.nbd.2005.12.006. 16455265

[jkae219-B2] Arbones ML , Thomazeau A, Nakano-Kobayashi A, Hagiwara M, Delabar JM. 2019. DYRK1A and cognition: a lifelong relationship. Pharmacol Ther. 194:199–221. doi:10.1016/j.pharmthera.2018.09.010. 30268771

[jkae219-B3] Bruser L , Bogdan S. 2017. Adherens junctions on the move—membrane trafficking of E-cadherin. Cold Spring Harb Perspect Biol. 9(3):a029140. doi:10.1101/cshperspect.a029140. 28096264 PMC5334258

[jkae219-B4] Cantor SB , Urano T, Feig LA. 1995. Identification and characterization of Ral-binding protein 1, a potential downstream target of Ral GTPases. Mol Cell Biol. 15(8):4578–4584. doi:10.1128/MCB.15.8.4578. 7623849 PMC230698

[jkae219-B5] Capdevila J , Guerrero I. 1994. Targeted expression of the signaling molecule decapentaplegic induces pattern duplications and growth alterations in Drosophila wings. EMBO J. 13(19):4459–4468. doi:10.1002/j.1460-2075.1994.tb06768.x. 7925288 PMC395378

[jkae219-B6] Cho B , Fischer JA. 2011. Ral GTPase promotes asymmetric Notch activation in the Drosophila eye in response to Frizzled/PCP signaling by repressing ligand-independent receptor activation. Development. 138(7):1349–1359. doi:10.1242/dev.056002. 21350007 PMC3114705

[jkae219-B7] Choi H , Larsen B, Lin ZY, Breitkreutz A, Mellacheruvu D, Fermin D, Qin ZS, Tyers M, Gingras AC, Nesvizhskii AI. 2011. SAINT: probabilistic scoring of affinity purification-mass spectrometry data. Nat Methods. 8(1):70–73. doi:10.1038/nmeth.1541. 21131968 PMC3064265

[jkae219-B8] Contreras EG , Egger B, Gold KS, Brand AH. 2018. Dynamic Notch signalling regulates neural stem cell state progression in the Drosophila optic lobe. Neural Dev. 13(1):25. doi:10.1186/s13064-018-0123-8. 30466475 PMC6251220

[jkae219-B9] Degoutin JL , Milton CC, Yu E, Tipping M, Bosveld F, Yang L, Bellaiche Y, Veraksa A, Harvey KF. 2013. Riquiqui and minibrain are regulators of the hippo pathway downstream of Dachsous. Nat Cell Biol. 15(10):1176–1185. doi:10.1038/ncb2829. 23955303 PMC4157670

[jkae219-B10] Douguet D , Raynaud J, Capderou A, Pannier C, Reiss G, Durand J. 1988. Muscular venous blood metabolites during rhythmic forearm exercise while breathing air or normoxic helium and argon gas mixtures. Clin Physiol. 8(4):367–378. doi:10.1111/j.1475-097X.1988.tb00280.x. 3409650

[jkae219-B11] Dowjat WK , Adayev T, Kuchna I, Nowicki K, Palminiello S, Hwang YW, Wegiel J. 2007. Trisomy-driven overexpression of DYRK1A kinase in the brain of subjects with Down syndrome. Neurosci Lett. 413(1):77–81. doi:10.1016/j.neulet.2006.11.026. 17145134 PMC1890010

[jkae219-B12] Egger B , Boone JQ, Stevens NR, Brand AH, Doe CQ. 2007. Regulation of spindle orientation and neural stem cell fate in the Drosophila optic lobe. Neural Dev. 2(1):1. doi:10.1186/1749-8104-2-1. 17207270 PMC1779784

[jkae219-B13] Egger B , Gold KS, Brand AH. 2010. Notch regulates the switch from symmetric to asymmetric neural stem cell division in the Drosophila optic lobe. Development. 137(18):2981–2987. doi:10.1242/dev.051250. 20685734 PMC2926952

[jkae219-B14] Fernandez-Martinez P , Zahonero C, Sanchez-Gomez P. 2015. DYRK1A: the double-edged kinase as a protagonist in cell growth and tumorigenesis. Mol Cell Oncol. 2(1):e970048. doi:10.4161/23723548.2014.970048. 27308401 PMC4905233

[jkae219-B15] García-Cerro S , Martínez P, Vidal V, Corrales A, Flórez J, Vidal R, Rueda N, Arbonés ML, Martínez-Cué C. 2014. Overexpression of Dyrk1A is implicated in several cognitive, electrophysiological and neuromorphological alterations found in a mouse model of Down syndrome. PLoS One. 9(9):e106572. doi:10.1371/journal.pone.0106572. 25188425 PMC4154723

[jkae219-B16] Guimera J , Casas C, Estivill X, Pritchard M. 1999. Human minibrain homologue (MNBH/DYRK1): characterization, alternative splicing, differential tissue expression, and overexpression in Down syndrome. Genomics. 57(3):407–418. doi:10.1006/geno.1999.5775. 10329007

[jkae219-B17] Guruharsha KG , Rual JF, Zhai B, Mintseris J, Vaidya P, Vaidya N, Beekman C, Wong C, Rhee DY, Cenaj O, et al 2011. A protein complex network of *Drosophila melanogaster*. Cell. 147(3):690–703. doi:10.1016/j.cell.2011.08.047. 22036573 PMC3319048

[jkae219-B18] Hakes AE , Otsuki L, Brand AH. 2018. A newly discovered neural stem cell population is generated by the optic lobe neuroepithelium during embryogenesis in *Drosophila melanogaster*. Development. 145(18):dev166207. doi:10.1242/dev.166207. 30254066 PMC6176933

[jkae219-B19] Hofbauer A , Campos-Ortega JA. 1990. Proliferation pattern and early differentiation of the optic lobes in *Drosophila melanogaster*. Rouxs Arch Dev Biol. 198(5):264–274. doi:10.1007/BF00377393. 28305665

[jkae219-B20] Holguera I , Desplan C. 2018. Neuronal specification in space and time. Science. 362(6411):176–180. doi:10.1126/science.aas9435. 30309944 PMC6368964

[jkae219-B21] Homem CC , Repic M, Knoblich JA. 2015. Proliferation control in neural stem and progenitor cells. Nat Rev Neurosci. 16(11):647–659. doi:10.1038/nrn4021. 26420377 PMC4667397

[jkae219-B22] Hong SH , Lee KS, Kwak SJ, Kim AK, Bai H, Jung MS, Kwon OY, Song WJ, Tatar M, Yu K. 2012. Minibrain/Dyrk1a regulates food intake through the Sir2-FOXO-sNPF/NPY pathway in Drosophila and mammals. PLoS Genet. 8(8):e1002857. doi:10.1371/journal.pgen.1002857. 22876196 PMC3410862

[jkae219-B23] Ikeda M , Ishida O, Hinoi T, Kishida S, Kikuchi A. 1998. Identification and characterization of a novel protein interacting with Ral-binding protein 1, a putative effector protein of Ral. J Biol Chem. 273(2):814–821. doi:10.1074/jbc.273.2.814. 9422736

[jkae219-B24] Ji J , Lee H, Argiropoulos B, Dorrani N, Mann J, Martinez-Agosto JA, Gomez-Ospina N, Gallant N, Bernstein JA, Hudgins L. 2015. DYRK1A haploinsufficiency causes a new recognizable syndrome with microcephaly, intellectual disability, speech impairment, and distinct facies. Eur J Hum Genet. 23(11):1473–1481. doi:10.1038/ejhg.2015.71. 25944381 PMC4613469

[jkae219-B25] Jörg DJ , Caygill EE, Hakes AE, Contreras EG, Brand AH, Simons BD. 2019. The proneural wave in the Drosophila optic lobe is driven by an excitable reaction-diffusion mechanism. Elife. 8:e40919. doi:10.7554/eLife.40919. 30794154 PMC6386523

[jkae219-B26] Jullien-Flores V , Mahé Y, Mirey G, Leprince C, Meunier-Bisceuil B, Sorkin A, Camonis JH. 2000. RLIP76, an effector of the GTPase Ral, interacts with the AP2 complex: involvement of the Ral pathway in receptor endocytosis. J Cell Sci. 113(16):2837–2844. doi:10.1242/jcs.113.16.2837. 10910768

[jkae219-B27] Kariya K , Koyama S, Nakashima S, Oshiro T, Morinaka K, Kikuchi A. 2000. Regulation of complex formation of POB1/epsin/adaptor protein complex 2 by mitotic phosphorylation. J Biol Chem. 275(24):18399–18406. doi:10.1074/jbc.M000521200. 10764745

[jkae219-B28] Kim SH , Cho JH, Park BO, Park BC, Kim JH, Park SG, Kim S. 2021. Phosphorylation of REPS1 at Ser709 by RSK attenuates the recycling of transferrin receptor. BMB Rep. 54(5):272–277. doi:10.5483/BMBRep.2021.54.5.266. 33407999 PMC8167248

[jkae219-B29] Kim H , Lee KS, Kim AK, Choi M, Choi K, et al 2016. A chemical with proven clinical safety rescues Down-syndrome-related phenotypes in through DYRK1A inhibition. Dis Model Mech. 9(8):839–848. doi:10.1242/dmm.025668. 27483355 PMC5007978

[jkae219-B30] Li X , Erclik T, Bertet C, Chen Z, Voutev R, Venkatesh S, Morante J, Celik A, Desplan C. 2013. Temporal patterning of Drosophila medulla neuroblasts controls neural fates. Nature. 498(7455):456–462. doi:10.1038/nature12319. 23783517 PMC3701960

[jkae219-B31] Litovchick L , Florens LA, Swanson SK, Washburn MP, DeCaprio JA. 2011. DYRK1A protein kinase promotes quiescence and senescence through DREAM complex assembly. Genes Dev. 25(8):801–813. doi:10.1101/gad.2034211. 21498570 PMC3078706

[jkae219-B32] Lowe SA , Usowicz MM, Hodge JJL. 2019. Neuronal overexpression of Alzheimer's disease and Down's syndrome associated DYRK1A/minibrain gene alters motor decline, neurodegeneration and synaptic plasticity in Drosophila. Neurobiol Dis. 125:107–114. doi:10.1016/j.nbd.2019.01.017. 30703437 PMC6419573

[jkae219-B33] Luco SM , Pohl D, Sell E, Wagner JD, Dyment DA, Daoud H. 2016. Case report of novel DYRK1A mutations in 2 individuals with syndromic intellectual disability and a review of the literature. BMC Med Genet. 17(1):15. doi:10.1186/s12881-016-0276-4. 26922654 PMC4769499

[jkae219-B34] Matsuzaki F , Shitamukai A. 2015. Cell division modes and cleavage planes of neural progenitors during mammalian cortical development. Cold Spring Harb Perspect Biol. 7(9):a015719. doi:10.1101/cshperspect.a015719. 26330517 PMC4563714

[jkae219-B35] Mishra AK , Bernardo-Garcia FJ, Fritsch C, Humberg TH, Egger B, Sprecher SG. 2018. Patterning mechanisms diversify neuroepithelial domains in the Drosophila optic placode. PLoS Genet. 14(4):e1007353. doi:10.1371/journal.pgen.1007353. 29677185 PMC5937791

[jkae219-B36] Morinaka K , Koyama S, Nakashima S, Hinoi T, Okawa K, Iwamatsu A, Kikuchi A. 1999. Epsin binds to the EH domain of POB1 and regulates receptor-mediated endocytosis. Oncogene. 18(43):5915–5922. doi:10.1038/sj.onc.1202974. 10557078

[jkae219-B37] Nakashima S , Morinaka K, Koyama S, Ikeda M, Kishida M, Okawa K, Iwamatsu A, Kishida S, Kikuchi A. 1999. Small G protein Ral and its downstream molecules regulate endocytosis of EGF and insulin receptors. EMBO J. 18(13):3629–3642. doi:10.1093/emboj/18.13.3629. 10393179 PMC1171441

[jkae219-B38] Neriec N , Desplan C. 2016. From the eye to the brain: development of the Drosophila visual system. Curr Top Dev Biol. 116:247–271. doi:10.1016/bs.ctdb.2015.11.032. 26970623 PMC5174189

[jkae219-B39] Ori-McKenney KM , McKenney RJ, Huang HH, Li T, Meltzer S, Jan LY, Vale RD, Wiita AP, Jan YN. 2016. Phosphorylation of beta-tubulin by the Down syndrome kinase, minibrain/DYRK1a, regulates microtubule dynamics and dendrite morphogenesis. Neuron. 90(3):551–563. doi:10.1016/j.neuron.2016.03.027. 27112495 PMC4860041

[jkae219-B40] Rammohan M , Harris E, Bhansali RS, Zhao E, Li LS, Crispino JD. 2022. The chromosome 21 kinase DYRK1A: emerging roles in cancer biology and potential as a therapeutic target. Oncogene. 41(14):2003–2011. doi:10.1038/s41388-022-02245-6. 35220406 PMC8977259

[jkae219-B41] Raveau M , Shimohata A, Amano K, Miyamoto H, Yamakawa K. 2018. DYRK1A-haploinsufficiency in mice causes autistic-like features and febrile seizures. Neurobiol Dis. 110:180–191. doi:10.1016/j.nbd.2017.12.003. 29223763

[jkae219-B42] Rosse C , L'Hoste S, Offner N, Picard A, Camonis J. 2003. RLIP, an effector of the Ral GTPases, is a platform for Cdk1 to phosphorylate epsin during the switch off of endocytosis in mitosis. J Biol Chem. 278(33):30597–30604. doi:10.1074/jbc.M302191200. 12775724

[jkae219-B43] Sawamoto K , Winge P, Koyama S, Hirota Y, Yamada C, Miyao S, Yoshikawa S, Jin MH, Kikuchi A, Okano H. 1999. The Drosophila Ral GTPase regulates developmental cell shape changes through the Jun NH(2)-terminal kinase pathway. J Cell Biol. 146(2):361–372. doi:10.1083/jcb.146.2.361. 10427090 PMC3206575

[jkae219-B44] Schindelin J , Arganda-Carreras I, Frise E, Kaynig V, Longair M, Pietzsch T, Preibisch S, Rueden C, Saalfeld S, Schmid B, et al 2012. Fiji: an open-source platform for biological-image analysis. Nat Methods. 9(7):676–682. doi:10.1038/nmeth.2019. 22743772 PMC3855844

[jkae219-B45] Schuldt AJ , Adams JH, Davidson CM, Micklem DR, Haseloff J, St Johnston D, Brand AH. 1998. Miranda mediates asymmetric protein and RNA localization in the developing nervous system. Genes Dev. 12(12):1847–1857. doi:10.1101/gad.12.12.1847. 9637686 PMC316902

[jkae219-B46] Shaikh MN , Gutierrez-Avino F, Colonques J, Ceron J, Hämmerle B, Tejedor FJ. 2016. Minibrain drives the Dacapo-dependent cell cycle exit of neurons in the Drosophila brain by promoting asense and prospero expression. Development. 143(17):3195–3205. doi:10.1242/dev.134338. 27510975

[jkae219-B47] Shaikh MN , Tejedor FJ. 2018. Mnb/Dyrk1A orchestrates a transcriptional network at the transition from self-renewing neurogenic progenitors to postmitotic neuronal precursors. J Neurogenet. 32(1):37–50. doi:10.1080/01677063.2018.1438427. 29495936

[jkae219-B48] Shannon P , Markiel A, Ozier O, Baliga NS, Wang JT, Ramage D, Amin N, Schwikowski B, Ideker T. 2003. Cytoscape: a software environment for integrated models of biomolecular interaction networks. Genome Res. 13(11):2498–2504. doi:10.1101/gr.1239303. 14597658 PMC403769

[jkae219-B49] Shard C , Luna-Escalante J, Schweisguth F. 2020. Tissue-wide coordination of epithelium-to-neural stem cell transition in the Drosophila optic lobe requires neuralized. J Cell Biol. 219(11):e202005035. doi:10.1083/jcb.202005035. 32946560 PMC7594497

[jkae219-B50] Shen CP , Jan LY, Jan YN. 1997. Miranda is required for the asymmetric localization of Prospero during mitosis in Drosophila. Cell. 90(3):449–458. doi:10.1016/S0092-8674(00)80505-X. 9267025

[jkae219-B51] Simoes S , Oh Y, Wang MFZ, Fernandez-Gonzalez R, Tepass U. 2017. Myosin II promotes the anisotropic loss of the apical domain during Drosophila neuroblast ingression. J Cell Biol. 216(5):1387–1404. doi:10.1083/jcb.201608038. 28363972 PMC5412560

[jkae219-B52] Szklarczyk D , Franceschini A, Wyder S, Forslund K, Heller D, Huerta-Cepas J, Simonovic M, Roth A, Santos A, Tsafou KP. 2015. STRING v10: protein-protein interaction networks, integrated over the tree of life. Nucleic Acids Res. 43(D1):D447–D452. doi:10.1093/nar/gku1003. 25352553 PMC4383874

[jkae219-B53] Tejedor FJ , Hammerle B. 2011. MNB/DYRK1A as a multiple regulator of neuronal development. FEBS J. 278(2):223–235. doi:10.1111/j.1742-4658.2010.07954.x. 21156027

[jkae219-B54] Tejedor F , Zhu XR, Kaltenbach E, Ackermann A, Baumann A, Canal I, Heisenberg M, Fischbach KF, Pongs O. 1995. Minibrain: a new protein kinase family involved in postembryonic neurogenesis in Drosophila. Neuron. 14(2):287–301. doi:10.1016/0896-6273(95)90286-4. 7857639

[jkae219-B55] Van Bon BW , Hoischen A, Hehir-Kwa J, De Brouwer AP, Ruivenkamp C, Gijsbers AC, Marcelis CL, De Leeuw N, Veltman JA, Brunner HG. 2011. Intragenic deletion in DYRK1A leads to mental retardation and primary microcephaly. Clin Genet. 79(3):296–299. doi:10.1111/j.1399-0004.2010.01544.x. 21294719

[jkae219-B56] Wang S , Chen X, Crisman L, Dou X, Winborn CS, Wan C, Puscher H, Yin Q, Kennedy MJ, Shen J. 2023. Regulation of cargo exocytosis by a Reps1-Ralbp1-RalA module. Sci Adv. 9(8):eade2540. doi:10.1126/sciadv.ade2540. 36812304 PMC9946360

[jkae219-B57] Wodarz A , Hinz U, Engelbert M, Knust E. 1995. Expression of crumbs confers apical character on plasma membrane domains of ectodermal epithelia of Drosophila. Cell. 82(1):67–76. doi:10.1016/0092-8674(95)90053-5. 7606787

[jkae219-B58] Yabut O , Domogauer J, D'Arcangelo G. 2010. Dyrk1A overexpression inhibits proliferation and induces premature neuronal differentiation of neural progenitor cells. J Neurosci. 30(11):4004–4014. doi:10.1523/JNEUROSCI.4711-09.2010. 20237271 PMC3842457

[jkae219-B59] Yang L , Paul S, Trieu KG, Dent LG, Froldi F, Forés M, Webster K, Siegfried KR, Kondo S, Harvey K, et al 2016. Minibrain and Wings apart control organ growth and tissue patterning through down-regulation of Capicua. Proc Natl Acad Sci U S A. 113(38):10583–10588. doi:10.1073/pnas.1609417113. 27601662 PMC5035877

[jkae219-B60] Yasugi T , Sugie A, Umetsu D, Tabata T. 2010. Coordinated sequential action of EGFR and Notch signaling pathways regulates proneural wave progression in the Drosophila optic lobe. Development. 137(19):3193–3203. doi:10.1242/dev.048058. 20724446

