# Supplementary material for: Regulation of Drosophila brain development and organ growth by the Minibrain/Rala signaling network
Source: G3 (Bethesda). 2024 Sep 13;14(11):jkae219. doi: 10.1093/g3journal/jkae219 (PMC11540318; doi:10.1093/g3journal/jkae219)
Supplement: jkae219_Supplementary_Data [file jkae219_supplementary_data.zip › Supplemental_Figures_G3-2024-405323.pdf]

Supplemental Figures S1-S8 for Brown et al., titled "Regulation of *Drosophila* brain development and organ growth by the Minibrain/Rala signaling network"

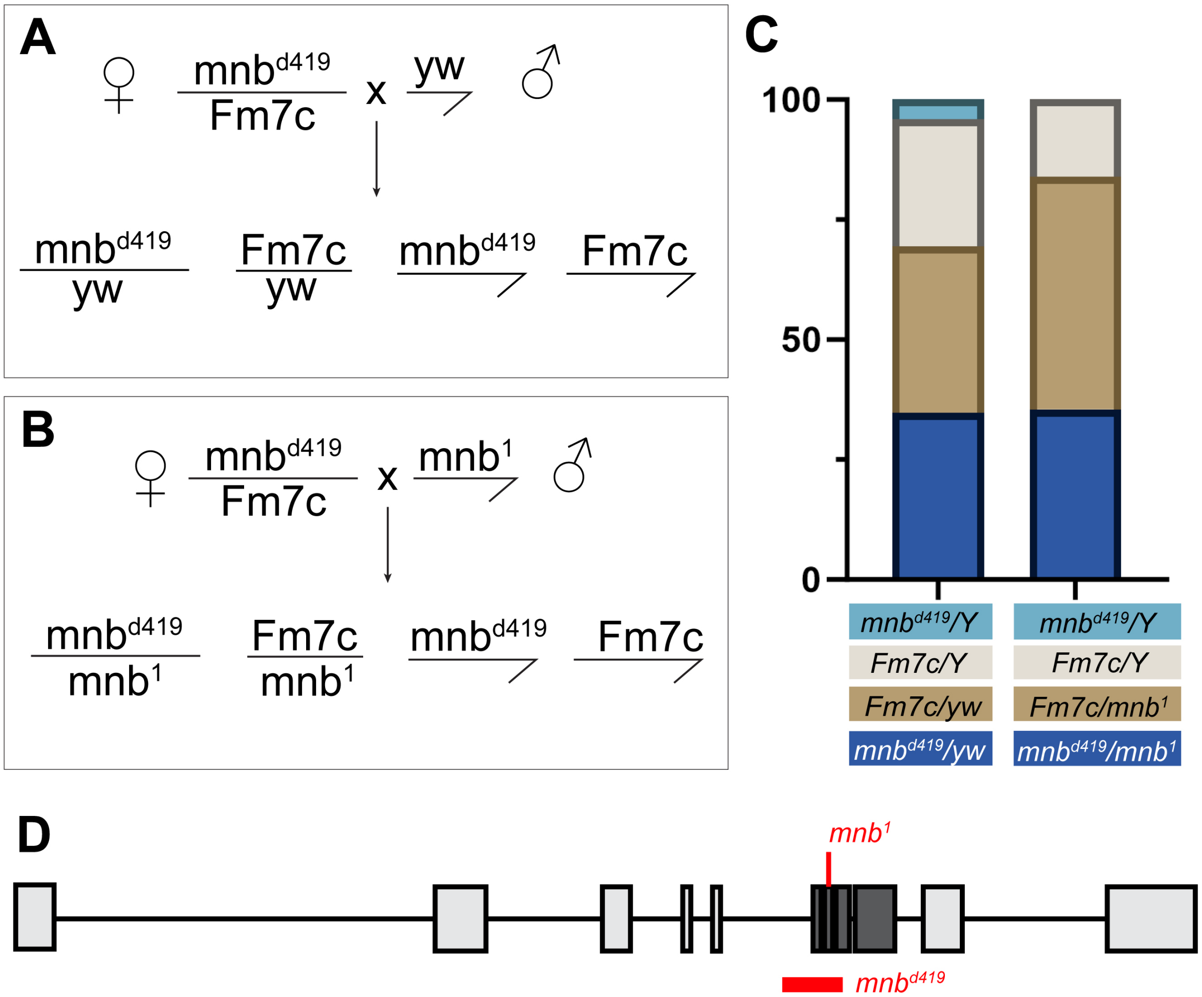

Figure S1. *mnb¹* complements *mnb^d419*. (A) Crossing scheme and expected progeny of *mnb^d419* females crossed with *yw* males. (B) Crossing scheme and expected progeny of *mnb^d419* females crossed with *mnb¹* males. (C) Distribution of observed progeny from crosses in A-B. (D) The nature of the *mnb^d419* allele (modified from (HONG *et al.* 2012)). Mnb exons are in light grey, kinase domain in dark grey. The *mnb¹* point mutation and the imprecise deletion in *mnb^d419* that deletes half of the kinase domain are marked in red.

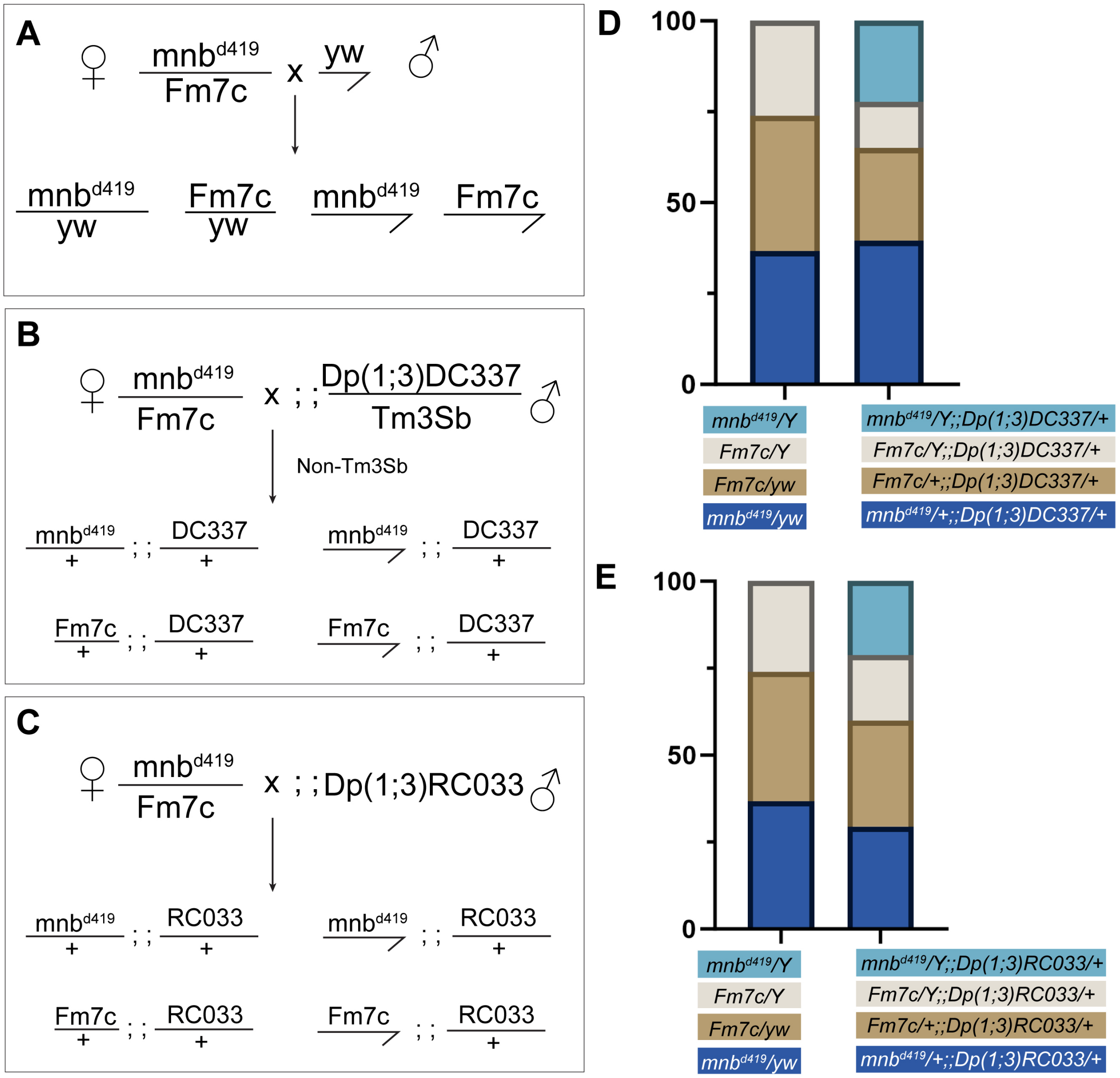

Figure S2. Duplications rescue *mnb^{d419}* lethality. (A) Crossing scheme and expected progeny of *mnb^{d419}* females crossed with *yw* males. (B) Crossing scheme and expected progeny of *mnb^{d419}* females crossed with *Dp(1;3)DC337* males. (C) Crossing scheme and expected progeny of *mnb^{d419}* females crossed with *Dp(1;3)RC033* males. (D) Distribution of observed progeny from crosses in (A-B). (E) Distribution of observed progeny from crosses in (A, C). Both duplications rescued the lethality associated with *mnb^{d419}*.

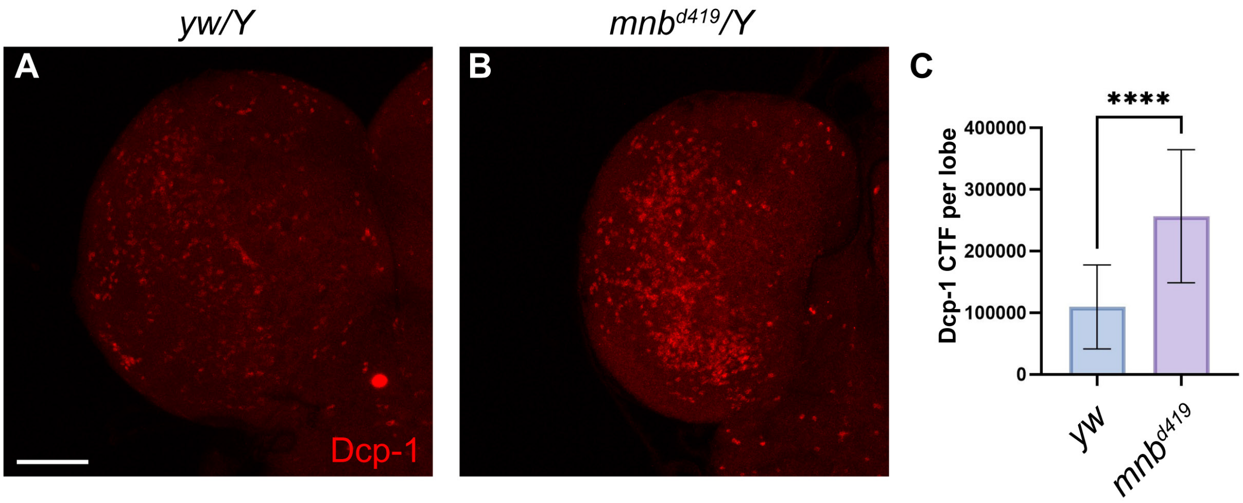

Figure S3. Loss of *mnb* increases apoptosis in the larval brain. (A-B) Confocal maximum intensity projections of brains from control *yw/Y* (A) and *mnb*^{d419}*/Y* (B) 3rd instar larvae immunostained for cleaved death caspase 1, Dcp-1 (red). Scale bar, 50 µm. (C) Quantification of cleaved Dcp-1 corrected total fluorescence (CTF) per optic lobe shown in (A-B). n=14, *****p*<0.0001, *p* value calculated using Student's t-test. Error bars indicate standard deviation.

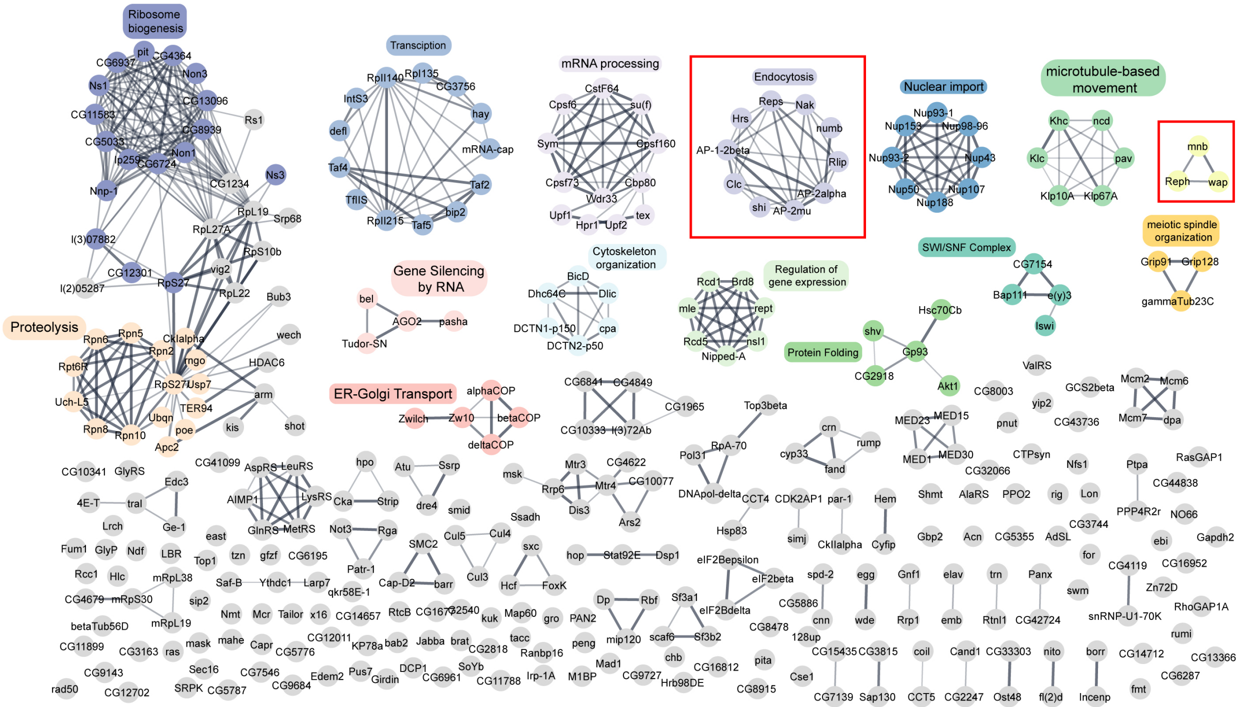

Figure S4. Mnb protein interaction network. Significantly enriched proteins identified in affinity purification-mass spectrometry (AP-MS) of *Drosophila* embryos expressing endogenously tagged Mnb. All proteins in this network were identified as high-confidence Mnb interactors in this analysis. Edges in this network were derived from the STRING protein database, and clusters were generated using the clusterMaker MCL Cluster app in Cytoscape. Gene Ontology analysis revealed biological process or cellular component enrichment terms, indicated near the respective clusters. Ribosome biogenesis, FDR=8.01E-21; proteolysis, FDR=9.25E-9; transcription, FDR=5.7E-12; mRNA processing, FDR=1.78E-8; endocytosis, FDR=1.64E-8; nuclear import, FDR=4.9E-13; microtubule-based movement FDR=6.6E-8; meiotic spindle organization, FDR=3.6E-5; SWI/SNF complex, FDR 2.66E-7; protein folding, FDR=3.9E-4; regulation of gene expression, FDR=4.4E-4; cytoskeleton organization, FDR=5.57E-6; ER-Golgi transport, FDR=2.3E-5; gene silencing, FDR=3.04E-6.

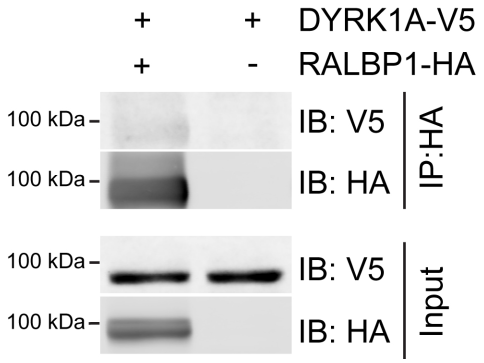

Figure S5. RALBP1 weakly interacts with DYRK1A. Co-immunoprecipitation of DYRK1A and RALBP1 in HEK293T cells through IP of RALBP1. IP: immunoprecipitation, IB: immunoblot.

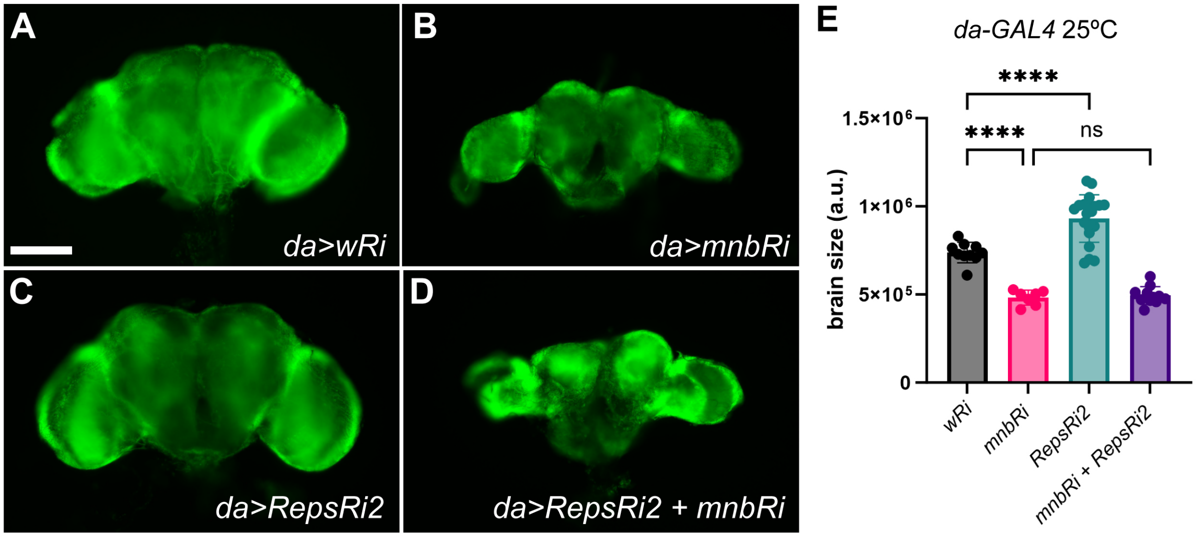

Figure S6. *mnb* is epistatic to *Reps* in brain growth assay with the *da-GAL4* driver. (A-D) Adult brains from female flies expressing the indicated RNAi transgenes using *da-GAL4*. (E) Quantification of brain size shown in (A-D) (n≥7 for each genotype). Brain size was measured as total area. DAPI signal shown in green. ****p<0.0001, p value calculated using ANOVA. Error bars represent SD. Scale bar, 100 μm.

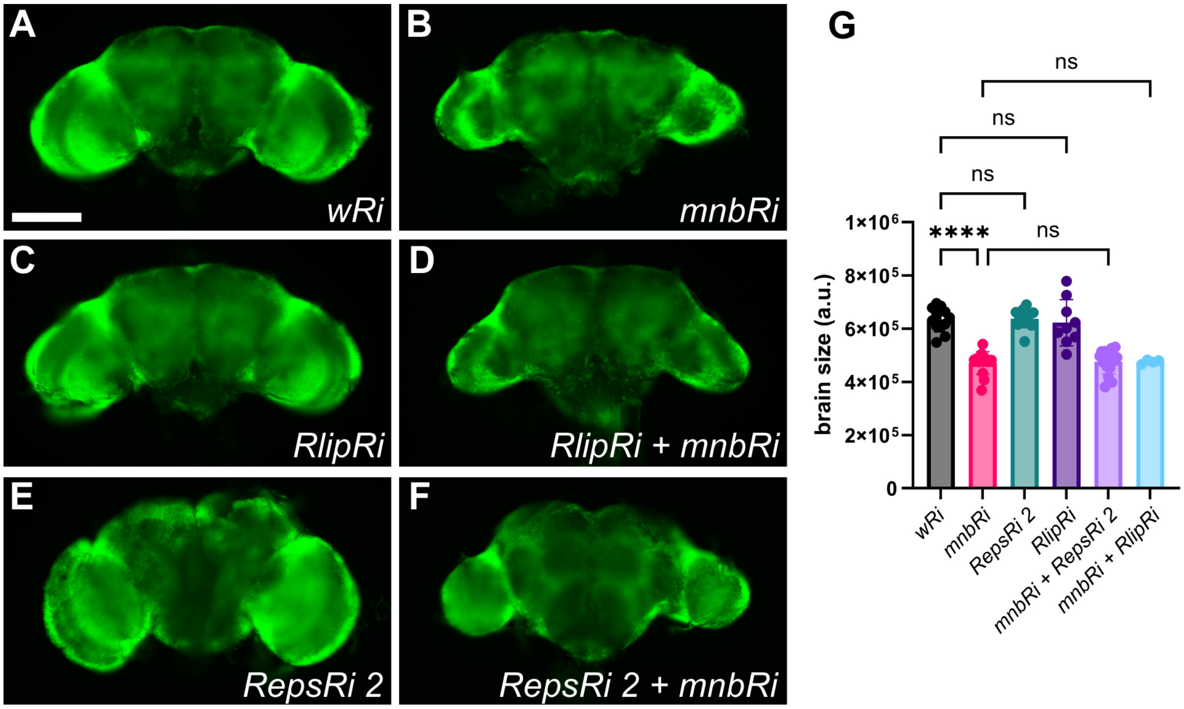

Figure S7. *mnb* is epistatic to *Reps* and *Rlip* in brain growth assay with the *c855a-GAL4* driver. (A-F) Adult brains from female flies expressing the indicated RNAi (*Ri*) transgenes using the NE-specific driver *c855a-GAL4*. (G) Quantification of brain size shown in (A-F) (n≥7 for each genotype). Brain size was measured as total area. DAPI signal shown in green. ****$p<0.0001$, p value calculated using ANOVA. Error bars represent SD. Scale bar, 100 µm.

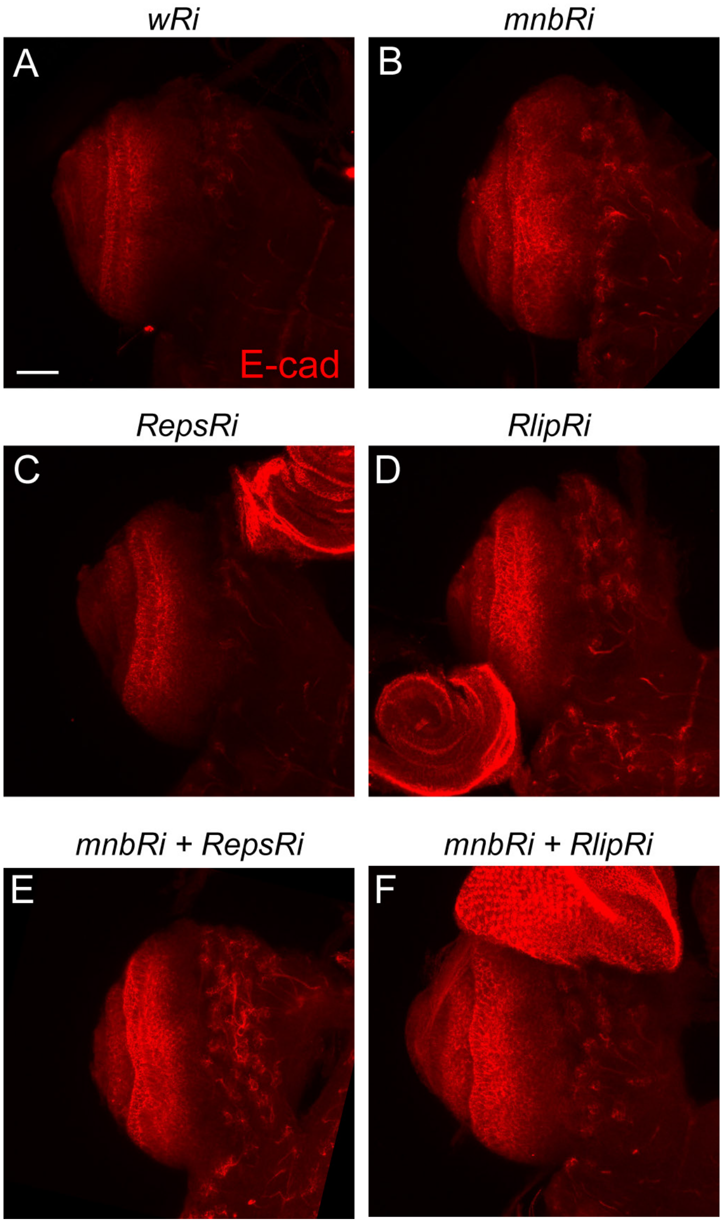

Figure S8. E-cad localization is not further disrupted by a joint knockdown of *mnb* with *Reps* and *Rlip* using *da-GAL4*. (A-F) Confocal maximum intensity projections of larval brains of the indicated genotypes (*Ri*, RNAi) immunostained for E-cad (red). Scale bar, 25 μm.
